# Derivation and characterization of ubiquitin-specific protease 18 inhibitors

**DOI:** 10.1172/jci.insight.202853

**Published:** 2026-05-19

**Authors:** Blessing O. Ogunlade, Kevin N. Dalby, Samuel C. Okpechi, Eun Jeong Cho, Liliya Tyutyunyk-Massey, Zibo Chen, Xiuxia Liu, Joseph Ivanic, Brian Luke, Shyamal D. Desai, Yair Alfaro, Ashwini K. Devkota, Rae M. Sammons, Gilbert G. Privé, Xi Liu, Ethan Dmitrovsky

**Affiliations:** 1 Molecular Pharmacology Program, Cancer Research Technology Program, Frederick National Laboratory for Cancer Research, Frederick, Maryland, USA.; 2 Targeted Therapeutic Drug Discovery and Development Program and; 3 Division of Chemical Biology and Medicinal Chemistry, The University of Texas at Austin, Austin, Texas, USA.; 4 Advanced Biomedical Computational Science, Frederick National Laboratory for Cancer Research, Frederick, Maryland, USA.; 5 Department of Biochemistry and Molecular Biology, Louisiana State University Health Science Center-School of Medicine, New Orleans, Louisiana, USA.; 6 School of Medicine and Health Sciences, Tecnologico de Monterrey, Monterrey, Mexico.; 7 Division of Cancer Genomics and Proteomics, Ontario Cancer Institute,; 8 Department of Medical Biophysics, and; 9 Department of Biochemistry, University of Toronto, Toronto, Ontario, Canada.

**Keywords:** Cell biology, Oncology, Lung cancer

## Abstract

Ubiquitin-Specific Protease 18 (USP18) is a deISGylation enzyme and antineoplastic target. To develop USP18 inhibitors, an enzymatically active human recombinant USP18 protein was engineered suitable for high-throughput screening of ~80,000 chemical compounds. Three of them substantially inhibited USP18 enzymatic activity, with β-lapachone having prominent antineoplastic activity. Independent β-lapachone treatments of murine and human lung cancer cell lines statistically significantly reduced proliferation and increased apoptosis. Gain of USP18 expression antagonized these effects. β-Lapachone treatments statistically significantly repressed lung cancer xenograft growth. β-Lapachone increased reactive oxygen species (ROS), but antineoplastic effects occurred at dosages with negligible ROS production. ROS scavenger treatments did not rescue β-lapachone effects at these concentrations, consistent with an ROS-independent mechanism. IFN-Stimulated Response Element (ISRE) reporter assays following β-lapachone treatment activated this reporter. USP18 cotransfection antagonized this activity. β-Lapachone treatments increased global ISGylation. RNA-seq of lung cancer cells engineered with or without enhanced USP18 expression showed specific pathways affected by β-lapachone treatment. Proteomic analysis of these treated cells revealed known and new ISGylated proteins. In silico modeling identified a unique USP18 pocket where these USP18 inhibitors bind. Engineered mutation of this pocket disrupted β-lapachone activity. Taken together, β-lapachone is an antineoplastic tool compound useful for USP18 inhibitor development.

## Abstract 2

β-lapachone was found to inhibit the deISGylase USP18. USP18 is a novel anti-cancer target. The molecular mechanisms of inhibition by β-lapachone are reported.

## Introduction

Lung cancer is the leading cause of cancer-related deaths (1). Despite advances in surgery, radiation therapy, chemotherapy, targeted therapy, and immunotherapy, 5-year survival for all lung cancer stages is only 28% (1). Most patients with lung cancer are diagnosed at stage IV, where the 5-year survival rate is far less than for all other stages (1, 2). While targeted treatments such as with EGFR, BRAF, and ALK inhibitors improved outcomes in subsets of patients with lung cancer, these therapies are active in only a minority of patients with lung cancer. Adding to the need for new antineoplastics in lung cancer is the frequent onset of resistance to current anticancer agents. These challenges indicate the need for novel strategies to improve lung cancer survival, as explored here.

Ubiquitination, a posttranslational modification, regulates diverse cellular processes, including protein stability, signal transduction, immune response, and other processes (3, 4). A related system involves ISGylation, an IFN-regulated pathway that modifies target proteins in a manner analogous to that of ubiquitination (5, 6). This pathway involves a set of enzymes that include E1-like activating enzyme (UBE1L) (7–9), E2-conjugating enzyme (UBCH8) (10, 11), and E3 ligase (HERC5A and others) (12, 13). Together, this pathway facilitates IFN-Stimulated Gene 15 (ISG15) conjugation to substrate proteins (14–16).

Ubiquitin-specific protease 18 (USP18) is the enzyme that deconjugates ISG15 from its substrates, reversing ISGylation (17, 18). Beyond its canonical role in IFN signaling, USP18 is implicated in tumorigenesis, where it stabilizes oncogenic proteins like PML/RARα, cyclin D1, and KRAS that in turn promote carcinogenesis (19–29). Thus, pharmacologic USP18 inhibitors are hypothesized to address a previously unmet need in oncology.

To address this knowledge gap, we developed an enzymatically active recombinant USP18 protein and performed a high-throughput screen to identify small-molecule inhibitors of the USP18 enzymatic activity. This and subsequent screens reported here identified β-lapachone as an USP18 inhibitor with anticancer activity.

β-Lapachone is a naturally occurring quinone product derived from the bark of Tabebuia avellanedae, with anticancer properties (30, 31). It is reported to exert its cytotoxic effects through NAD(P)H:quinone oxidoreductase-1–mediated (NQO1-mediated) redox cycling, generating reactive oxygen species (ROS) that cause DNA damage and necrosis (32–34). Preclinical studies revealed that β-lapachone preferentially targets NQO1-overexpressing tumors, including lung, breast, pancreatic, and prostate cancers (33–37).

β-Lapachone repressed growth of colorectal cancer cells through ferroptosis (38) and suppressed cervical cancer via interaction with AKT1 (39). β-Lapachone was shown to induce apoptosis, inhibit proliferation, and enhance the efficacy of chemotherapeutic agents in cancer cells (40–43). β-Lapachone has been evaluated in early-phase clinical trials. A phase I study (NCT00054977) in patients with advanced solid tumors determined the safety profile, pharmacokinetics, and maximum tolerated dose. Combined regimens with chemotherapy and radiation are being studied in preclinical and clinical settings. Despite its potential antineoplastic effects, β-lapachone was not previously functionally validated as a USP18 inhibitor.

We report here that β-lapachone treatment statistically significantly reduced proliferation and induced apoptosis in murine and human lung cancer cell lines. Engineered USP18 overexpression opposed these effects. In vivo β-lapachone treatment statistically significantly repressed tumor growth in a human lung cancer cell line xenograft transplanted into immunodeficient mice. β-Lapachone pharmacodynamically increased ROS levels, but its antineoplastic effects occurred at concentrations where ROS accumulation was hardly detected. Notably, ROS scavenger treatments did not prevent β-lapachone anticancer effects at these dosages. This indicated that a mechanism other than ROS production was engaged by β-lapachone treatment.

An IFN-stimulated response element (ISRE) reporter assay found that β-lapachone activated JAK/STAT signaling, as expected for USP18 inhibition. β-Lapachone treatment of lung cancer cells was found to enhance global ISGylation. RNA-seq after β-lapachone treatment (as compared with controls) of lung cancer cells identified expressed genes and pathways preferentially regulated by β-lapachone treatment. The cotransfection of USP18 provided insights into the pathways regulated by USP18 inhibition via β-lapachone treatment. Proteomic analysis identified known and novel ISG15-conjugated proteins. That β-lapachone acts through specific USP18 residues and within a defined USP18 pocket was shown through computational modeling and functional analyses of mutant USP18 species.

In summary, the findings presented here establish β-lapachone as a previously unrecognized functional USP18 inhibitor and as a valuable tool for examining USP18-mediated oncogenic signaling. β-Lapachone itself serves as a proof-of-concept tool compound for elucidation of future derivatives with even greater potency as USP18 inhibitors. The identification of β-lapachone as a USP18 inhibitor opens potentially new ways to target USP18 in cancer or immune-based therapy.

## Results

### High-throughput screen identifies candidate USP18 inhibitors.

**Figure 1:**
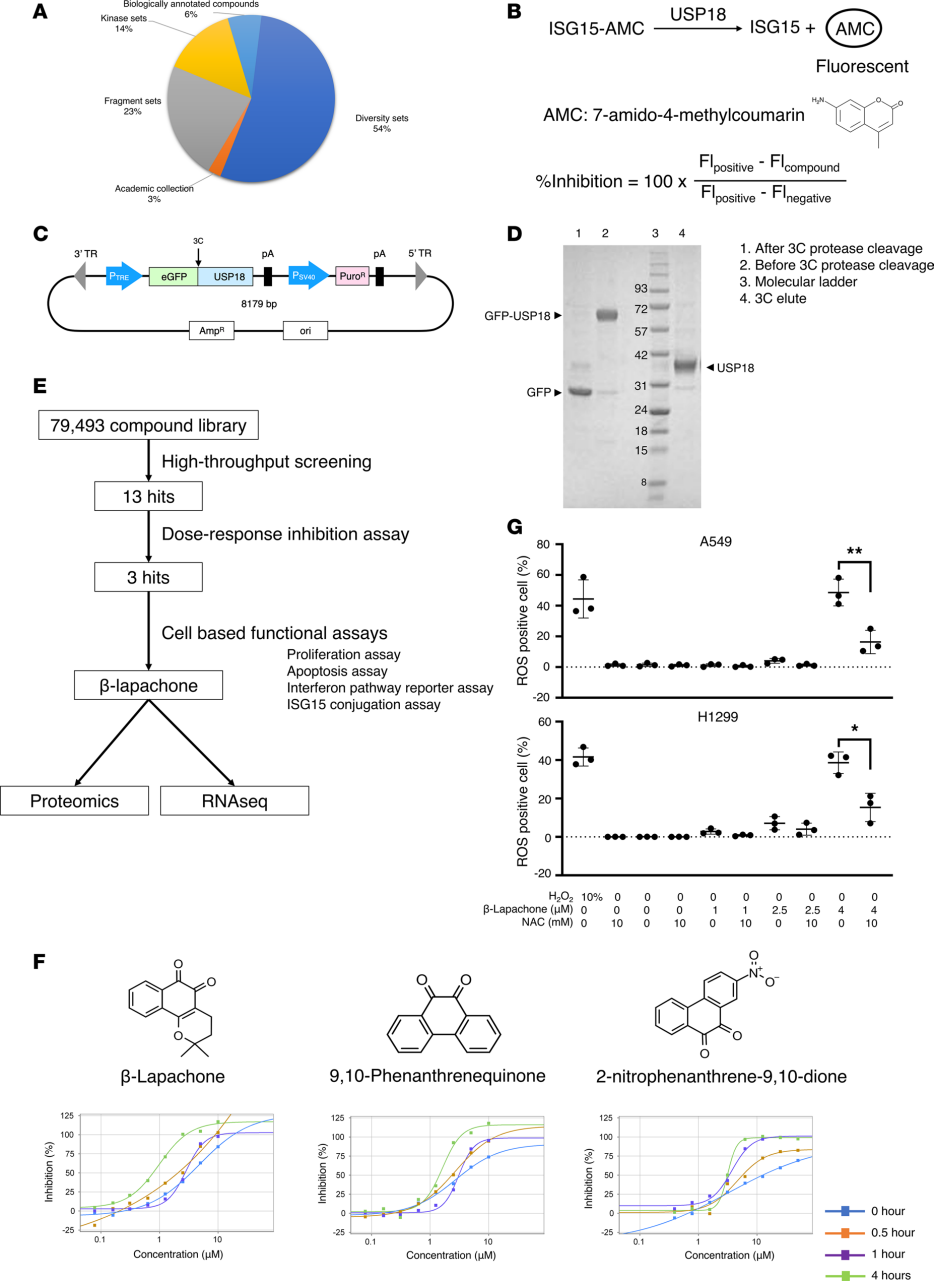
High-throughput screen identifies candidate USP18 small molecule inhibitors. (**A**) The features of the 79,493-compound library used for high-throughput screening are displayed, showing the proportions of fragment, kinase, diversity, biologically annotated, and academic compound sets that were interrogated. (**B**) The diagram of the fluorogenic enzymatic assay used to identify small-molecule USP18 inhibitors is shown. Recombinant, enzymatically active recombinant USP18 deconjugated ISG15-AMC substrate, and fluorescence intensity was used to calculate the percentage inhibition of each tested compound. (**C**) Schematic representation of the GFP-tagged USP18 expression vector used to generate enzymatically active USP18 protein. (**D**) Immunoblot analysis of USP18 species before and after 3C protease cleavage confirmed the production of the enzymatically active USP18 used for the biochemical assays. (**E**) Workflow of the screening strategy indicated the progression from primary screening to hit validation, including the selection of 13 primary hits, 3 reproducible USP18-inhibitory compounds, and identification of β-lapachone as the lead compound for further evaluation. (**F**) Dose-response inhibition curves of β-lapachone, 9,10-phenanthrenequinone, and 2-nitrophenanthrene-9,10-dione are displayed using the same fluorogenic enzymatic assay described in **B**. (**G**) The percent of ROS production was measured in human A549 and H1299 lung cancer cell lines following β-lapachone treatment at the indicated concentrations with or without cotreatment with the ROS scavenger N-acetylcysteine (NAC, 10 mM). Two-tailed Student’s *t* tests compared differences between arms with a *P* < 0.05 deemed statistically significant. Data are shown as mean ± SD with the symbols indicating *P < 0.05 and **P < 0.01, respectively.

To identify small-molecule inhibitors of USP18, high-throughput screening was performed using a diverse compound library containing 79,493 small molecules, as shown in Figure 1A. A fluorescence-based enzymatic assay was established using recombinant, enzymatically active USP18 protein as displayed in Figure 1, B and C. Purified USP18 displayed deISGylating activity, which was independently confirmed by immunoblot assays in Figure 1D. Primary screening, followed by counter screening, identified 13 candidate inhibitors that were subjected to dose–response assays at various treatment durations (0 hour, 0.5 hour, 1 hour, and 4 hours).

The strategy deployed in this screen is summarized in Figure 1E. This approach progressed from high-throughput enzymatic assays through multiple biochemical, transcriptional, and cell-based assays. Five compounds displayed reproducible enzymatic inhibition against USP18 in concentration- and time-dependent manners. The increased inhibition observed with prolonged pretreatment suggests that these compounds may act via a noninstantaneous or potentially covalent mechanism, and the detailed mechanism of action is under investigation. Among the 5 candidate inhibitors, 3 compounds (β-lapachone; 9,10-phenanthrenequinone; and 2-nitrophenanthrene-9,10-dione) shared similar structural scaffolds and physicochemical properties, prompting further interest in their mode of action, as shown in Figure 1F. Two of these 5 compounds did not appreciably suppress lung cancer cell proliferation and were not moved forward for further screening, as shown in Supplemental Figure 1 (supplemental material available online with this article; https://doi.org/10.1172/jci.insight.202853DS1). The observation of similar scaffolds in the 3 most active USP18 inhibitors motivated the subsequent molecular modeling studies to investigate how these compounds, particularly β-lapachone, engaged USP18 at functional and structural levels.

β-Lapachone is reported to confer cytotoxic effects through NQO1-mediated redox cycling, generating ROS that cause DNA damage and necrosis (32–34). However, the data displayed in Figure 1G found that β-lapachone treatment generated negligible ROS at 1 μM and 2.5 μM concentrations. A marked increase in ROS was only observed at the higher dosage of β-lapachone (4 μM), as measured by flow cytometry. Notably, combined treatment with the ROS scavenger N-acetylcysteine (NAC) substantially attenuated this ROS elevation, at the β-lapachone 4 μM dosage, but hardly at the lower dosages as shown in Figure 1G. These findings indicated that β-lapachone was activating anticancer mechanisms at concentrations lower than 4 μM that did not engage ROS. Studies were next conducted to elucidate these mechanisms.

### β-Lapachone treatment suppresses lung cancer cell proliferation.

**Figure 2:**
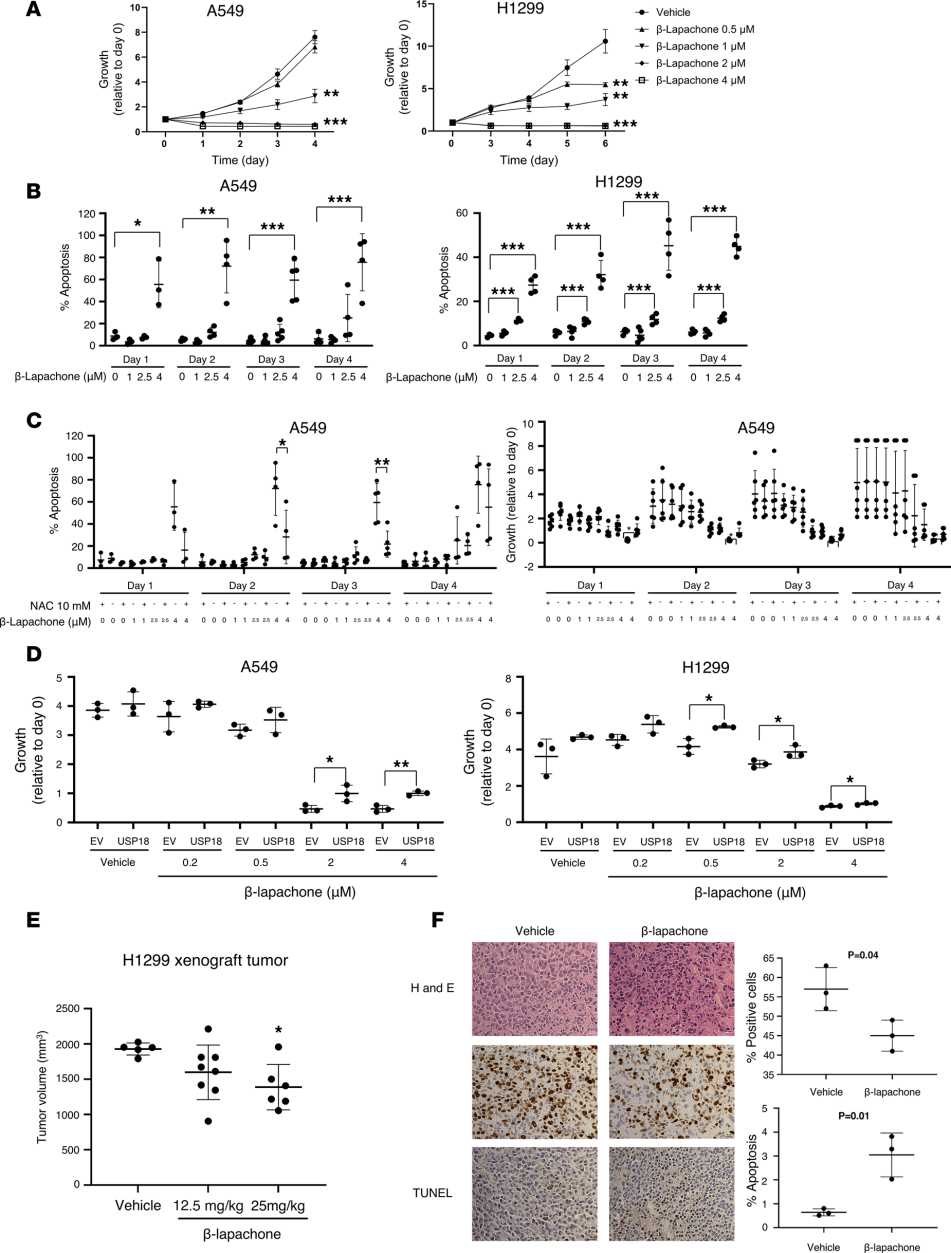
β-Lapachone reduces lung cancer cell proliferation and increases apoptosis. (**A** and **B**) Proliferation and apoptosis assays of human lung cancer A549 and H1299 lung cancer cell lines following β-lapachone treatment at the indicated concentrations are displayed. β-Lapachone treatment statistically significantly reduced proliferation and augmented apoptosis in a dose-dependent manner as compared with vehicle controls. (**C**) Apoptosis and proliferation were displayed for A549 lung cancer cells following β-lapachone treatment (versus vehicle treatment) at the indicated concentrations with or without cotreatment with the ROS scavenger N-acetylcysteine (NAC, 10 mM). β-Lapachone increased apoptosis significantly at the 4 μM dosage but not at lower concentrations. NAC cotreatment statistically significantly antagonized this effect only at the 4 μM dosage. (**D**) Growth suppression by β-lapachone treatment was also antagonized by engineered gain of USP18 expression. β-Lapachone treatment repressed proliferation of A549 and H1299 lung cancer cells transfected with an empty vector (EV). In contrast, USP18 overexpression partially reversed this growth inhibition at the 2 μM and 4 μM dosages. (**E**) In vivo antitumor activity of β-lapachone treatment is displayed at the left panel (12.5 mg/kg or 25 mg/kg body weight) as evaluated using the H1299 human lung cancer xenograft model. The 25 mg/kg body weight of β-lapachone statistically significantly reduced tumor growth as compared with vehicle-treated controls after 29 days of treatment. (**F**) Representative H&E staining, Ki-67 immunostaining, and apoptosis scoring are displayed for different treatment groups. Two-tailed Student’s *t* tests compared differences between study groups with a *P* value below 0.05 deemed statistically significant. Data are shown as mean ± SD. *P < 0.05, **P < 0.01, and ***P < 0.001.

Functional consequences of β-lapachone treatment were explored. β-Lapachone statistically significantly repressed proliferation of human lung cancer cell lines A549 and H1299 (Figure 2A) in a dose-dependent manner. β-Lapachone treatment augmented apoptosis in A549 and H1299 cells, as shown in Figure 2B. This growth inhibition was not reversed by cotreatment with the ROS scavenger NAC, as seen in Figure 2C. The β-lapachone treatment at 2.5 μM concentrations also increased apoptosis in these cancer cells, but NAC cotreatment did not appreciably attenuate this response, as displayed in Figure 2C. These results suggest that the antiproliferative and proapoptotic activities of β-lapachone occur at lower concentrations largely through an ROS-independent mechanism. In addition, β-lapachone suppressed proliferation and induced apoptosis of murine lung cancer 344SQ cells in a similar dose-dependent pattern as in human lung cancer cells, as shown in Supplemental Figure 2.

To explore if these effects are mediated through USP18, USP18 rescue experiments were performed. Independently engineered gain of USP18 expression in human A549 and H1299 lung cancer cells antagonized β-lapachone–induced growth inhibition (Figure 2D). This indicates that reduction of USP18 activity contributed to the antiproliferative signals of β-lapachone.

To confirm and extend these in vitro findings to the in vivo setting, human lung cancer xenograft models were interrogated using the H1299 lung cancer cell line. Oral administration of β-lapachone at the 12.5 mg/kg and 25 mg/kg of mouse body weight decreased lung tumor growth as compared with controls. The tumor growth decline at the 25 mg/kg of mouse body weight reached statistical significance versus vehicle controls (Figure 2E). Notably, this decline in lung cancer growth was associated with a statistically significant reduction in proliferation as measured by Ki-67 staining and an increase in apoptosis TUNEL scoring, as displayed in Figure 2F. These findings demonstrate that β-lapachone treatments (as compared with vehicle control treatments) confer anticancer effects in immunodeficient mice harboring human lung cancer cells. Given these antineoplastic effects, it was next studied whether these actions were mediated through USP18 inhibition.

### β-Lapachone antagonizes USP18-dependent activities.

**Figure 3:**
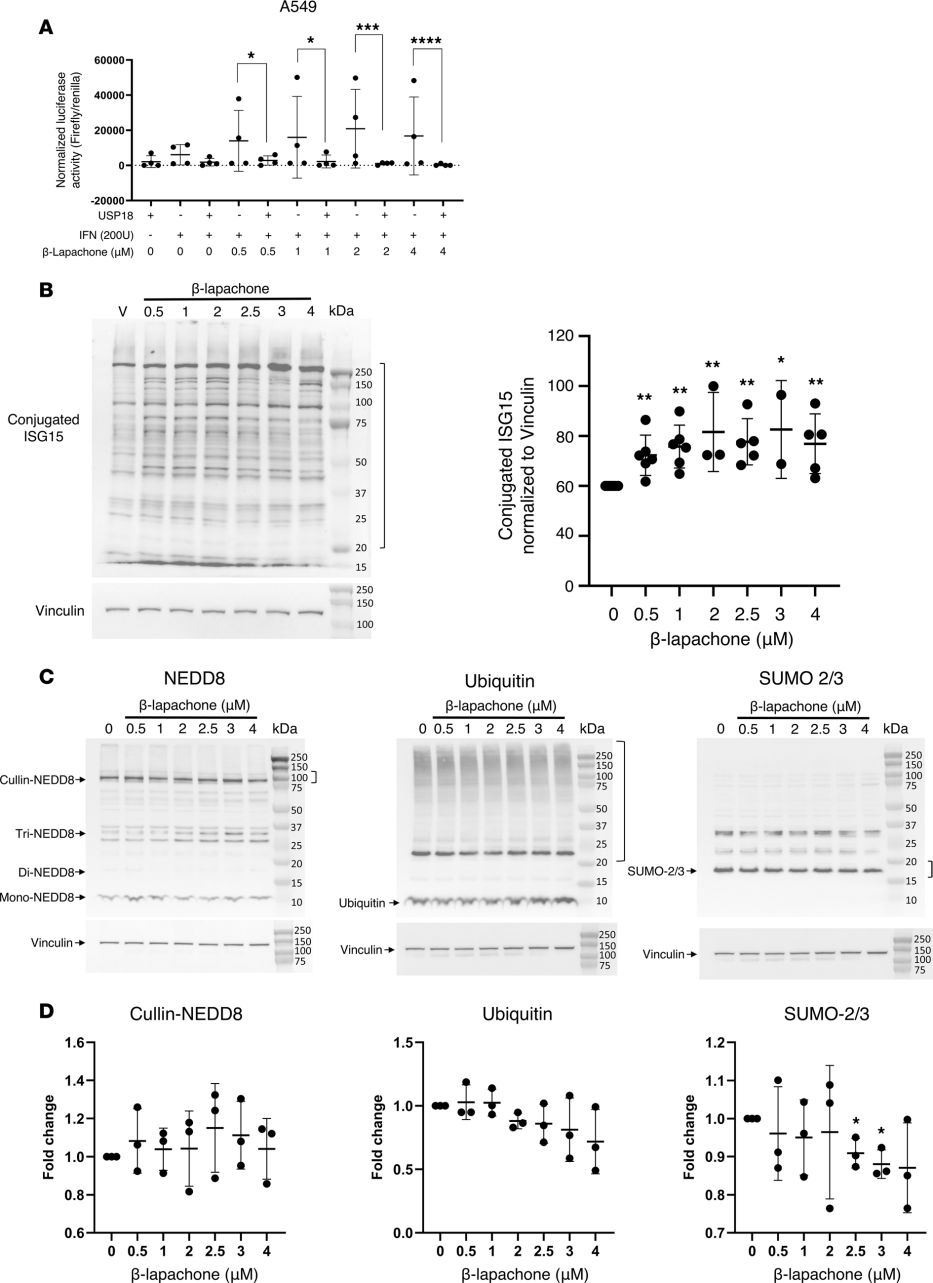
β-Lapachone antagonizes USP18-dependent activity. (**A**) IFN-stimulated response element (ISRE) reporter assays were performed to evaluate the effect of β-lapachone treatment on IFN pathway activation. A549 lung cancer cells were transiently transfected with an insertless vector (EV) as a control or a USP18 expression vector and treated with the indicated concentrations of β-lapachone with or without IFN (200 units) cotreatment. β-Lapachone treatment statistically significantly increased ISRE reporter activity in a concentration-dependent manner, whereas USP18 overexpression antagonized this activation. (**B**) Immunoblot analyses were performed to assess the effects of β-lapachone treatment on global ISGylation in A549 cells. Representative immunoblot images and the quantification of conjugated ISG15 species within the indicated bracketed region of 4 independent experiments are displayed. A statistically significant increase in global ISGylation was evident after dose-dependent β-lapachone treatments. (**C**) Independent immunoblot analyses of NEDD8, ubiquitin, and SUMO-2/3 conjugation after β-lapachone treatment as compared with vehicle control were performed to evaluate pathway selectivity. β-Lapachone treatment did not confer detectable changes in ubiquitin or NEDD8 conjugation. Only a modest change in SUMO-2/3 conjugation was observed at the highest β-lapachone concentration. The vinculin signals present in the middle and right panels were from the same immunoblot and lysate that were independently probed for ubiquitin and SUMO-2/3. The NEDD8 immunoblot was from a different lysate. These immunoblots present representative signals for each of these species. (**D**) Quantification of the NEDD8, ubiquitin, and SUMO-2/3 conjugates shown in **C** was performed using 3 independent replicate experiments. Two-tailed Student’s *t* tests compared differences between study groups with a *P* value below 0.05 deemed statistically significant. Data are shown as mean ± SD. *P < 0.05, **P < 0.01, ***P < 0.001 and ****P < 0.0001.

To confirm target specificity, it was explored whether β-lapachone treatment affected USP18-dependent signaling in A549 lung cancer cells. Consistent with an antagonist of USP18 function, β-lapachone treatment enhanced activity in a dose-dependent manner of the transfected ISRE reporter assay, as depicted in Figure 3A. In contrast, engineered gain of human USP18 expression repressed this ISRE activity. This inhibited the increase in reported activity that was conferred by β-lapachone treatment. Consistent with these findings, immunoblot analysis demonstrated that β-lapachone treatment also increased global ISGylation in A549 human lung cells, as seen in Figure 3B. Notably, β-lapachone treatment preferentially affected ISGylation, given that this treatment had little effect on ubiquitin, SUMO-2/3, or NEDD8 conjugation other than a minor effect on SUMO-2/3 conjugation observed only at the highest β-lapachone examined treatment concentrations (Figure 3, C and D).

### Molecular modeling of β-lapachone binding to a USP18.

**Figure 4:**
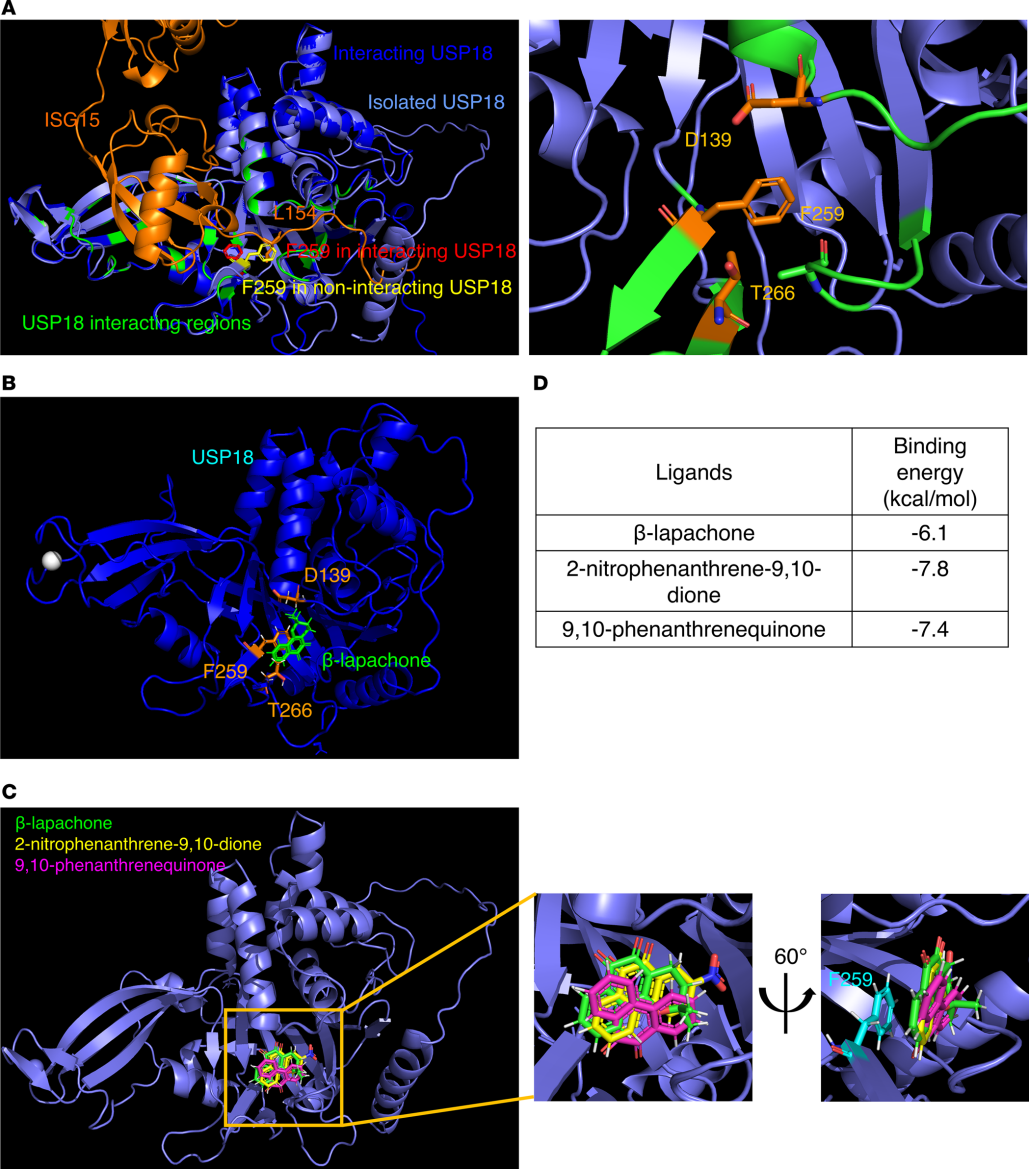
Molecular modeling of β-lapachone binding to USP18. (**A**) Structural modeling of USP18 and ISG15 complex formation identified a hydrophobic pocket in USP18 required for substrate engagement. Within this pocket, 3 key amino acid residues — D139, F259, and T266 were found to mediate ISG15 binding. (**B**) Molecular docking analysis revealed that β-lapachone (green) interacts with USP18 at the identified hydrophobic pocket, with the critical residues D139, F259, and T266 highlighted in brown. (**C**) Docking simulations of β-lapachone; 9,10-phenanthrenequinone; and 2-nitrophenanthrene-9,10-dione showed that all 3 compounds occupy this same hydrophobic pocket of USP18 in a similar spatial configuration. (**D**) Calculated binding energies for β-lapachone; 9,10-phenanthrenequinone; and 2-nitrophenanthrene-9,10-dione are individually displayed, supporting comparable affinities for binding to USP18 protein.

To elucidate the molecular basis of USP18 inhibition by individual β-lapachone, 9,10-phenanthrenequinone, and 2-nitrophenanthrene-9,10-dione treatments, molecular modeling of the USP18–ISG15 interaction interface was performed. Intriguingly, structural analyses identified a hydrophobic pocket needed for substrate engagement. Within this pocket, the phenylalanine residue F259 was found to be a critical determinant of ligand binding. As shown in Figure 4A, the conformation of F259 differs between ISG15-bound (interacting) and unbound (noninteracting) USP18, indicating its dynamic role in mediating substrate recognition. Additional modeling highlighted 3 residues — D139, F259, and T266 — as key interaction points through which β-lapachone engages USP18 within this hydrophobic pocket (Figure 4B).

Consistent with these findings, docking analyses revealed the compounds having USP18 enzymatic inhibitory properties (β-lapachone, 9,10-phenanthrenequinone, and 2-nitrophenanthrene-9,10-dione) bound the identified USP18 pocket in a similar manner, as displayed in Figure 4C. Binding energy calculations supported these findings, revealing favorable energetics for ligand engagement, with β-lapachone exhibiting a calculated binding energy of approximately –6.1 kcal/mol, comparable with those of 9,10-phenanthrenequinone and 2-nitrophenanthrene-9,10-dione, as shown in Figure 4D. These results provide a mechanistic basis through which β-lapachone and its analogs inhibit USP18 enzymatic activity through occupancy of a conserved hydrophobic binding pocket defined by residues D139, F259, and T266.

**Figure 5:**
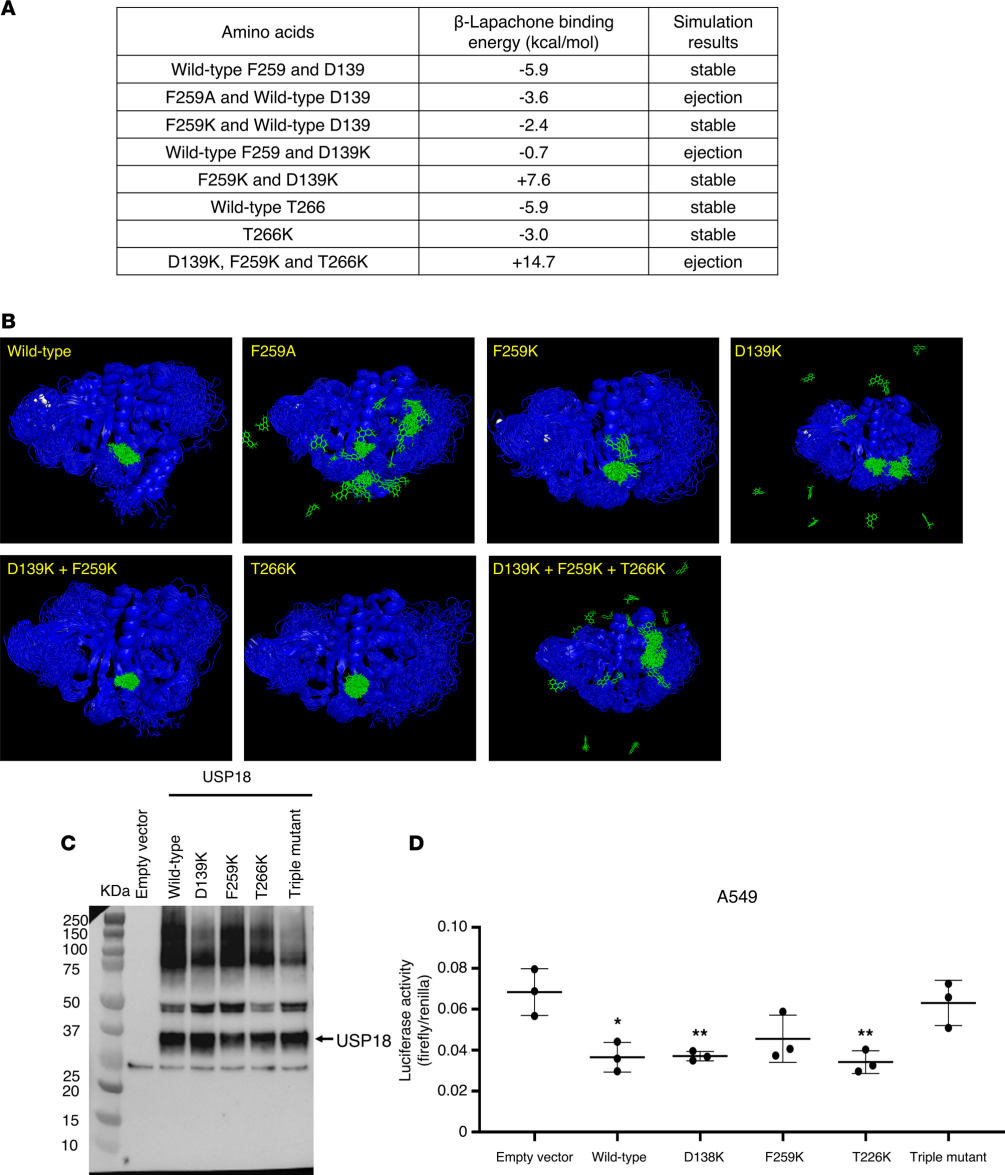
Mutational analyses validate the USP18 residues critical for β-lapachone binding and activity. (**A**) Binding energy calculations and 1,000 nanosecond molecular dynamics simulations were performed to evaluate the effects of single (D139K, F259K, T266K), double, and triple amino acid substitutions on β-lapachone–USP18 interactions. Mutation of any of the 3 key residues markedly reduced predicted binding affinity, with the triple mutant (D139K/F259K/T266K) abolishing stable binding. (**B**) Molecular dynamics visualization of β-lapachone (green) bound to WT and mutant USP18 proteins (blue) after 1,000 nanosecond simulations showed stable binding to WT USP18 but with ejection of β-lapachone from mutant USP18 species. (**C**) Immunoblot analysis confirmed comparable expression of WT and mutant USP18 proteins following transfection of each construct, including single and triple mutants. (**D**) The A549 lung cancer cell line was transfected with an ISRE luciferase reporter plasmid together with either empty vector, WT USP18, or USP18 constructs harboring individual (D139K, F259K, T266K) or triple mutations at the indicated residues. Luciferase activity was measured 48 hours after transfection and normalized to Renilla luciferase. Two-tailed Student’s *t* tests compared differences between study groups with a *P* value below 0.05 deemed statistically significant. Data are shown as mean ± SD. *P < 0.05 and **P < 0.01.

To independently validate these molecular modeling predictions, site-directed mutagenesis of the 3 critical amino acid residues (D139, F259, and T266) in USP18 was next performed. In silico energy calculations revealed that mutation of any of these residues would markedly reduce the predicted binding affinity between β-lapachone and USP18, with the greatest loss of interaction observed in examination of the triple mutant (Figure 5A and Supplemental Figure 3). Structural visualization confirmed that β-lapachone bound tightly to WT USP18 but did not similarly bind mutant USP18 proteins, consistent with loss of the hydrophobic-pocket interaction displayed in Figure 5B.

To ensure proper protein expression, immunoblot analyses verified comparable expression levels of WT and mutant USP18 constructs in Figure 5C. Functional testing using the ISRE reporter assay showed that IFN treatment elevated the luciferase activity, while transfection of WT USP18 species into A549 lung cancer cells opposed this activation. In marked contrast, transfection of the triple mutant USP18 species did not appreciably affect this activation and indicated that the triple mutant USP18 species was no longer functional, as displayed in Figure 5D. Together, these findings provide a mechanistic basis through which β-lapachone disrupts USP18 enzymatic activity by binding the hydrophobic pocket needed for ISG15 recognition (44).

Among the 3 highlighted USP18 candidate inhibitors, β-lapachone was prioritized for further pharmacodynamic studies because it exhibited the most robust inhibitory activity in enzymatic, biochemical, cellular, and tumorigenicity assays. β-Lapachone is a natural product with known antineoplastic properties (35–37). It is a tool compound to further assess the consequences of USP18 inhibition.

### Transcriptomic profiling and USP18-dependent β-lapachone response.

**Figure 6:**
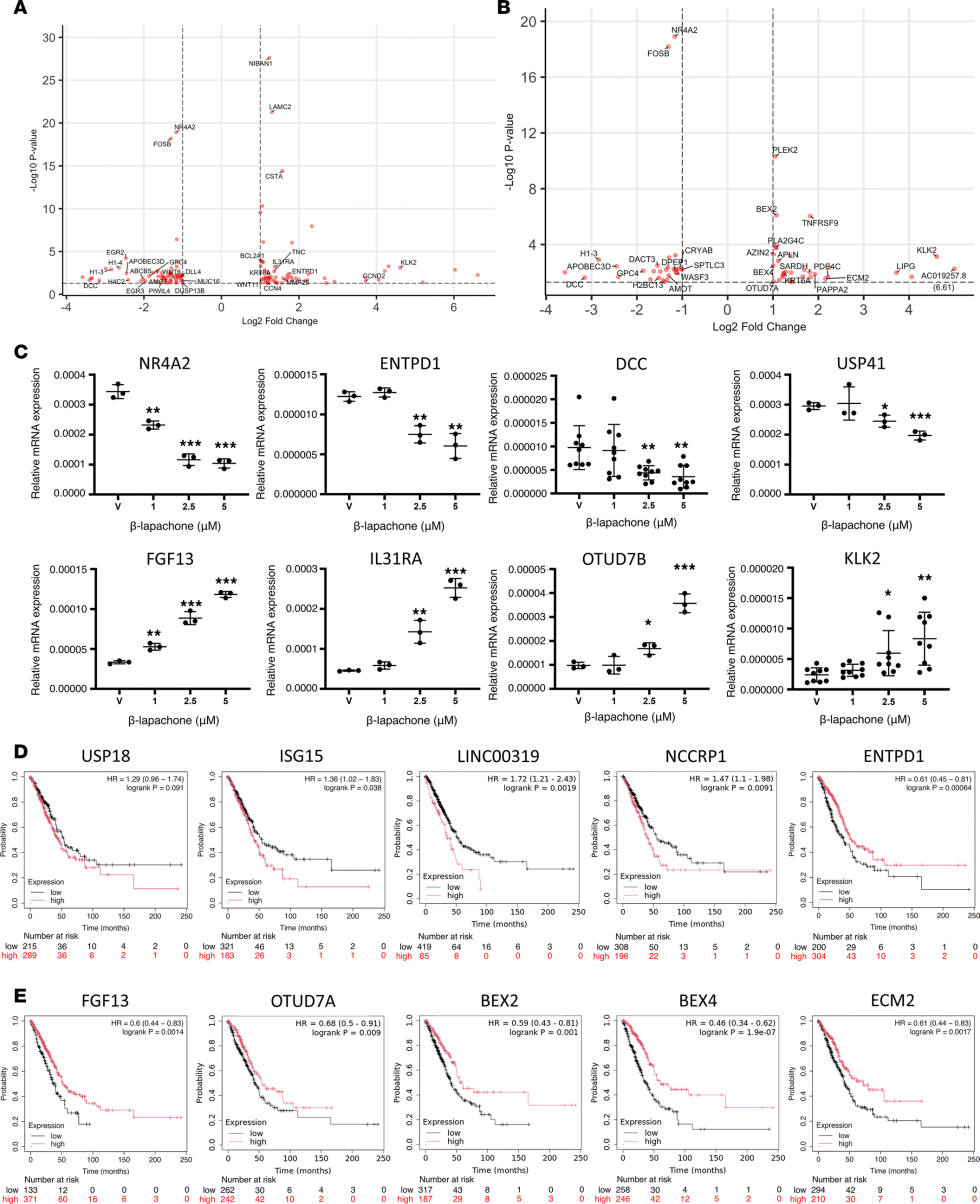
Transcriptomic profiling identifies USP18-dependent gene expression changes conferred by β-lapachone treatment relative to vehicle control. (**A**) Volcano plot displayed the differentially expressed genes in A549 human lung cancer cells treated with β-lapachone (2.5 μM) as compared with vehicle-treated controls. β-Lapachone treatment caused distinct patterns of gene upregulation and downregulation. (**B**) Volcano plot of A549 lung cancer cells engineered to overexpress USP18 versus empty vector controls is shown following β-lapachone treatment (2.5 μM) as compared with vehicle control. Genes whose expression changes were opposed by USP18 overexpression represent β-lapachone regulated transcripts that are USP18 dependent. (**C**) Quantitative real-time PCR assays validated representative β-lapachone–regulated genes identified in **B**, including those decreased (NR4A2, ENTPD1, DCC, and USP41) and those increased (FGF13, IL31RA, OTUD7A, and KLK2) after β-lapachone treatment. (**D**) Representative β-lapachone treatment repressed expression of USP18-dependent genes, including LINC00319, NCCRP1, and ENTPD1. Differential expression profiles of many of species showed statistically significant associations with overall survival in TCGA lung adenocarcinoma cohort. (**E**) Representative β-lapachone treatment increased expression of USP18-regulated genes, including FGF13, IL31RA, OTUD7A, and KLK2, exhibited statistically significant associations with improved overall survival in the same TCGA cohort. Two-tailed Student’s *t* tests compared differences between study groups with a *P* value below 0.05 deemed statistically significant. Data are shown as mean ± SD. *P < 0.05, **P < 0.01 and ***P < 0.001. Survival analyses for **D** and **E** were performed using the Kaplan-Meier method using the log-rank test with a *P* value below 0.05 deemed statistically significant.

RNAseq was next performed to determine transcriptional pathways engaged by β-lapachone treatment. This treatment of A549 lung cancer cells as compared with vehicle controls caused a distinct pattern of differentially expressed genes, including both upregulated and downregulated transcripts (Figure 6A and Supplemental Figure 4A). To determine whether these responses were mediated by USP18 inhibition, transcriptomes of USP18-overexpressing A549 lung cancer cells were compared with that of empty-vector controls treated with β-lapachone. Exogenous USP18 expression antagonized a subset of β-lapachone–induced transcriptional changes, indicating that the effects displayed in Figure 6B and Supplemental Figure 4, B and C, are likely USP18 dependent.

Real-time PCR assays independently validated representative expressed genes that were either reduced (NR4A2, ENTPD1, DCC, and USP41) or increased (FGF13, IL31RA, OTUD7A, and KLK2) following β-lapachone treatment (Figure 6C). To assess the translational relevance of these β-lapachone–regulated and USP18-dependent genes, transcriptomic and survival data were interrogated using The Cancer Genome Atlas (TCGA) lung adenocarcinoma cohort. High mRNA expression levels of USP18 or ISG15 were each associated with poorer overall lung cancer survival in Figure 6D. The prognostic value of USP18 and ISG15 in lung squamous cell carcinoma is shown in Supplemental Figure 4D. Among β-lapachone–suppressed genes whose expression was rescued by USP18 overexpression, including LINC00319, NCCRP1, and ENTPD1, higher expression in human lung adenocarcinomas was associated with unfavorable survival outcomes in Figure 6D.

In contrast, β-lapachone–expressed genes that were antagonized by engineered USP18 overexpression, including FGF13, IL31RA, OTUD7A, and KLK2, showed the opposite survival patterns with higher expression levels in lung adenocarcinomas that were associated with an improved survival (Figure 6E). These findings serve to identify USP18-dependent gene expression pathways that are engaged by β-lapachone treatment. To delineate species that could mediate the antineoplastic effects of β-lapachone, proteomic analyses were next done.

### Proteomic analyses reveal β-lapachone–regulated ISG15-related proteins.

**Figure 7:**
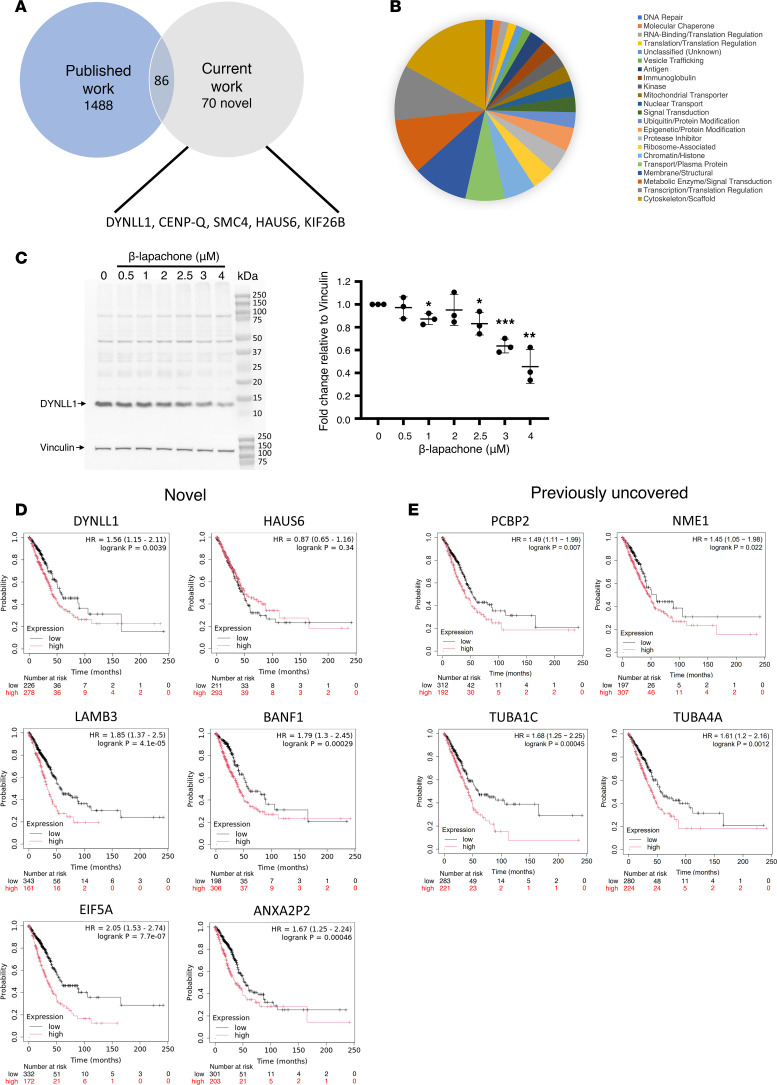
Proteomic analyses reveal β-lapachone treatment affected ISG15-related proteins. (**A**) ISG15 immunoprecipitation followed by mass spectrometry proteomics analysis was performed in A549 lung cancer cells that expressed an empty vector or a USP18 expression construct, and these transfectants were treated with β-lapachone (2.5 μM) or vehicle treatments. Comparative proteomic analyses identified potential ISG15-conjugated proteins whose levels were increased by β-lapachone and rescued by engineered USP18 overexpression. Among the ISG15-conjugated targets detected, 86 were previously reported ISG15 substrates and 70 were found as new ISG15-conjugated proteins. (**B**) Functional categorization of the β-lapachone treatment arm that regulated ISG15 conjugates revealed broad distribution across multiple cellular processes. (**C**) Immunoblot validation of the representative ISG15-conjugated protein, DYNLL1, confirmed that its expression was regulated by β-lapachone–treatment relative to vehicle controls (quantification is shown). Data are shown as mean ± SD. *P < 0.05, **P < 0.01, and ***P < 0.001. (**D**) TCGA lung adenocarcinoma cohort analyses demonstrated that several β-lapachone upregulated ISG15-conjugated proteins, including DYNLL1, HAUS6, LAMB3, BANF1, EIF5A, and ANXA2P2, were statistically significantly associated with lung cancer survival outcomes. (**E**) Four previously unrecognized ISG15-conjugated proteins, PCBP2, NME1, TUBA1C, and TUBA4A, were confirmed as β-lapachone regulated ISG15 targets whose elevated expression predicted an unfavorable survival in TCGA lung adenocarcinoma cases. Survival analysis for **D** and **E** highlighted species was performed using the Kaplan-Meier method, using the log-rank test. A *P* value below 0.05 was deemed statistically significant.

To further define the consequences of USP18 inhibition, we performed ISG15 immunoprecipitation–mass spectrometry analyses in A549 lung cancer cells expressing an empty vector or a USP18-expressing vector. These transfectants were each treated with vehicle or β-lapachone at the 2.5 μM concentration. Under stringent elution conditions designed to retain preferentially the covalently bound ISG15 conjugates, β-lapachone treatment substantially affected the ISG15-conjugated proteome. Comparison between β-lapachone–treated control cells and USP18-overexpressing A549 lung cancer transfectants identified proteins whose ISGylation were rescued by USP18 expression, as expected for effects that were USP18-dependent. Among the ISG15-conjugated proteins identified, 86 matched previously reported ISG15 targets, while 70 represented new candidate ISG15-conjugated proteins, as displayed in Figure 7A and Supplemental Figure 5. Pathway enrichment analysis revealed that these β-lapachone–regulated ISG15 substrates were distributed across multiple cellular processes, including signal transduction, RNA and protein modification, mitochondrial transport, vesicle trafficking, and chromatin regulation, as shown in Figure 7B.

The expression of a representative potentially novel ISGylated protein, DYNLL1, was interrogated by immunoblot analyses following β-lapachone as compared with vehicle treatment. These findings are shown in Figure 7C and reveal a β-lapachone dose-dependent repression of DYNLL1 immunoblot expression. To establish the translational relevance of this observation, TCGA lung adenocarcinoma cohort was examined for the effect on survival of differential expression of DYNLL1. Intriguingly, a favorable survival outcome was observed in lung cancer cases having low as compared with high levels of DYNLL1 levels within the lung cancers. Other novel and previously recognized ISGylated species were examined for their associations for lung cancer survival in Figure 7, D and E. Differential expression profiles of several identified ISG15-conjugated proteins were explored for their prognostic effect across different cancer types, as shown in Supplemental Figure 6.

Several β-lapachone–upregulated ISG15 substrates, including DYNLL1, HAUS6, LAMB3, BANF1, EIF5A, and ANXA2P2 showed statistically significant associations with survival of patients with cancer, as seen in Figure 7D. Four previously unrecognized ISG15 targets (PCBP2, NME1, TUBA1C, and TUBA4A) were confirmed as β-lapachone–regulated conjugates whose high expression profiles were associated with unfavorable survival outcomes in Figure 7E. These observations broaden an understanding of β-lapachone treatment effects on the ISGylated proteome.

## Discussion

Lung cancer is the leading cause of cancer mortality, and new therapeutic strategies are needed to improve clinical outcomes (1). Recent studies have highlighted the deISGylation enzyme USP18 as an emerging antineoplastic target, due to its central role in regulating type I IFN signaling through deISGylation from substrate proteins (3–9, 20–23). That prior work indicated that USP18-mediated deISGylation can stabilize multiple proteins involved in immune modulation as well as those affecting cancer cell survival, proliferation, and tumorigenicity.

Building upon this previous work, this study identified and characterized USP18 inhibitors that exhibit antineoplastic effects. Of these compounds, β-lapachone was found to be a small-molecule USP18 antagonist that exhibited the most prominent antineoplastic properties in lung cancer models in in vitro and in vivo assays. Pharmacological USP18 inhibitors with antineoplastic activity are not yet reported having functional anticancer validation. That is why this study addresses a prior knowledge gap in cancer research.

A high-throughput screen of ~80,000 small-molecule compounds assessed enzymatically active recombinant human USP18 to identify potential small molecule inhibitors. These studies yielded 42 candidate compounds with USP18 inhibitory activity. To explore further the USP18 inhibitory effects of these compounds, cell-based and lung cancer xenograft assays were performed, along with transactivation, transcriptional, and proteomic assays. Three compounds emerged from these studies as candidates exhibiting the most USP18 inhibitory activities.

Molecular docking and modeling analyses revealed that all 3 highlighted compounds were bound to the same hydrophobic pocket of USP18 protein. This is consistent with previous structural studies that revealed this pocket mediated USP18’s interaction with ISG15 (44). Interestingly, all 3 compounds showed structural convergence. Two of these 3 compounds shared a similar structural backbone. These findings indicate a common pharmacophore. This provides a basis for future structure-guided compound optimization. Supporting this interpretation were studies performed in Figures 4 and 5, which showed that, based on modeling studies, a mutated form of USP18 was engineered to no longer bind to β-lapachone. As expected, this deISGylase did not function as did the WT USP18 species in IFN transactivation assays in Figure 5D. Prior work compared the relationship between the enzymatic and scaffolding properties of USP18 species (45). Perhaps β-lapachone binding to USP18 elicited conformational changes within human USP18 protein that affected the ISRE reporter assay results reported here.

The high hydrophobicity of these candidate compounds made conduct of biophysical ligand–protein binding assays difficult to perform. This is why studies reported here relied on molecular docking and modeling to infer binding interactions. Future work should identify optimal lead compounds to generate drug-like USP18 inhibitors. Those studies would be facilitated by in silico modeling studies as presented here that could accelerate this development.

Functional studies that were performed in lung cancer cells demonstrated that β-lapachone exhibited prominent USP18-inhibitory activity. Treatment with β-lapachone increased global ISGylation and activated ISRE reporters, consistent with loss of USP18-mediated deISGylation. It was thus not surprising that cotransfection of WT but not the mutant form of USP18 that lacked the ability to sustain this binding pocket antagonized the activity of β-lapachone. These β-lapachone antineoplastic effects were associated with reduced cell proliferation and increased apoptosis in both human and murine lung cancer cells. Engineered overexpression of WT but not triple mutant USP18 species antagonized β-lapachone effects. These findings are consistent with the antineoplastic activity of β-lapachone engaging the enzymatic activity of USP18.

One hypothesized feature of the β-lapachone mechanism of action is its ability to generate ROS via NQO1-mediated redox cycling (32). It is notable that the data presented in Figures 1 and 2 show that effective dosages of β-lapachone that yielded prominent anticancer effects produced minimal ROS levels. Likewise, treatment with ROS scavengers did not appreciably reverse the observed β-lapachone antineoplastic signals that were observed at dosages lower than 4 μM. These findings indicate that β-lapachone can act through USP18 inhibition in the experiments performed here, rather than via ROS-dependent mechanisms, as expected if NQO1-mediated redox cycling were prominently engaged at this dosage. Future structural modification or optimization of β-lapachone analogs should aim to enhance USP18 specificity while minimizing ROS-inducing activity to improve selectivity and therapeutic anticancer properties.

Prominent in vivo antitumor activity of β-lapachone was observed. Treatment of the H1299 xenograft lung cancer model led to statistically significant lung cancer growth inhibition in this in vivo model, as shown in Figure 2. This antineoplastic effect was associated with a decrease in proliferation and an increase in apoptosis markers within β-lapachone–treated lung tumor bearing mice. These findings confirm and extend prior work showing statistically significant in vivo anticancer effects of β-lapachone treatment of A549 lung cancer xenografts as well as in other in vivo cancer xenograft models (37, 38, 43, 46). These studies provide a therapeutic rationale for targeting USP18 in lung or other cancers that overexpress USP18 relative to the histopathologically normal lung (23). That β-lapachone treatment acted at least partly by altering IFN signaling was consistent with the transcriptomic and proteomic analyses displayed in Figures 6 and 7. Those studies revealed that β-lapachone can upregulate IFN-stimulated genes and broaden the repertoire of ISGylated proteins, including previously unrecognized ISG15 substrates.

These results indicate that the ISG15/USP18 axis could regulate broader cellular pathways than previously reported. Identification of these previously unrecognized ISGylated targets should elucidate novel proteins that are engaged by this posttranslational modification and that would elicit antitumorigenic effects. Likewise, these species might inform translationally relevant biomarker development following USP18 antagonism.

Beyond its role in regulating ISGylation and IFN signaling, USP18 has additional IFN-independent functions that may contribute to cancer biology and influence therapeutic responses (17). The transcriptomic and proteomic changes observed following β-lapachone treatment were not limited to canonical IFN-stimulated genes but also involved pathways linked to signal transduction, metabolism, vesicle trafficking, and protein regulation, indicating that USP18 inhibition can affect multiple cellular processes. These broader effects suggest that pharmacologic targeting of USP18 may have consequences that extend beyond IFN pathway modulation. At the same time, USP18 plays an important role in immune regulation, and its inhibition could influence immune responses depending on cellular and tissue context. While the present study focused on tumor-intrinsic effects using immunodeficient xenograft models, future studies in immunocompetent systems will be necessary to define the immune consequences and therapeutic window of USP18 inhibition.

The findings presented here establish β-lapachone as a USP18 inhibitor. However, β-lapachone is at best a tool compound and not yet a clinical lead, given its off-target activities or drug-like properties. The structural convergence of the 3 most active USP18 inhibitors described here provides a rationale for the future discovery of a clinical lead compound. These studies should aim to develop derivatives of β-lapachone or other promising compounds with related scaffolds that can interact with the USP18 pocket described here and have optimal selectivity, potency, and formulation needed for a promising clinical lead.

In summary, this study found that β-lapachone antagonizes USP18 antineoplastic activity. This reduced proliferation, increased apoptosis, and augmented ISGylation. Collectively, this led to repressed human lung cancer growth in mice as in Figure 2 and in lung and other in vivo cancer models that were reported by others (37, 38, 43, 45). Anticancer function of a USP18 inhibitor is just one aspect of the actions of USP18 (19–29). USP18 also plays a role in regulation of IFN signaling and viral infections (17–19). Thus, any USP18 inhibitor would likely have therapeutic properties beyond its antineoplastic activity.

Prior studies revealed that USP18 is an appealing antineoplastic target (19–29). The studies presented here indicate that β-lapachone elicits substantial inhibition of USP18 activity. This finding, coupled with the USP18 pocket through which β-lapachone and other USP18 antagonists bind, provide avenues to develop more effective inhibitors in future work. The USP18 inhibitory compounds described here will help develop future USP18 inhibitors with desired drug-like properties. Such compounds that exhibit enhanced USP18 expression could enter clinical trial testing for lung and likely other cancers.

## Methods

### Sex as a biological variable.

This study examined antineoplastic effects of β-lapachone in female NSG mice as recipients for H1299 lung cancer xenografts because female recipient mice typically exhibit less aggressive behaviors than do male mice. Studies performed in xenograft lung tumors did not reveal an appreciable difference in antitumor responses to β-lapachone observed between male and female recipient mice (40–43).

### Compound libraries.

A diverse library of 79,493 small-molecule compounds was screened. This library comprised fragment libraries (18,037 compounds; ChemBridge and ChemDiv), kinase-focused libraries (11,240 compounds), diversity libraries (42,957 compounds; National Cancer Institute, ChemDiv, LifeChem, and Maybridge/Thermo Fisher Scientific), biologically annotated compounds (5,175 compounds), and an academic collection (2,000 unique compounds from the University of Texas at Austin and the University of Kansas). Compounds were dissolved in dimethyl sulfoxide (DMSO) at 10 mM concentrations, placed into 384-well plates, and stored at –40°C.

### Screening for USP18 inhibitors and validation.

A biochemical assay monitoring the deISGylation activity of USP18 was performed using the fluorogenic substrate 7-amido-4-methylcoumarin (AMC) labeled ISG15 (#UL-553, Bio-techne) and was deployed as a high-throughput small molecule screening and hit characterization (47). Primary screening was conducted at 50 μM compound concentrations following this procedure: 5 μL of enzyme mixture containing 2X USP18 in 1X assay buffer (50 mM HEPES pH 7.5, 10 mM DTT, 5% v/v glycerol, 0.01% (v/v) Tween-20, and 0.1% (w/v) BSA) or 1X buffer only were dispensed to 384-well assay plates (#262260, Thermo Fisher Scientific). Using 50nL of compounds (Columns 3–22) or 100% DMSO (columns 1, 2, 23, and 24) were dispensed to assay plates and incubated for 1 hour at room temperature. Then 5 μL of 2X ISG15-AMC in 1X buffer was added to start the reaction. The reaction at room temperature was quenched after 1 hour by adding 5 μL of 0.1N HCl. The fluorescence intensity at excitation/emission wavelengths of 360 ± 20 nm/450 ± 20 nm was read on a Synergy H4 multifunctional plate reader (Agilent). Final concentrations of mUSP18 and ISG15-AMC were 25 nM and 600 nM, respectively. Fluorescence interference was assessed on primary hits using the following control. An AMC-only counter screen was performed in which hit compounds (50 μM) were incubated with free AMC fluorophore (600 nM) in the absence of enzyme under matched buffer/DMSO conditions, and fluorescence was measured at Ex**/**Em 360/450 nm. Compounds that reduced the AMC signal by more than 30% were deprioritized.

A dose-response inhibition of each compound was examined using the same biochemical assay. Compounds at various concentrations, starting from 50 μM and serially diluted 8 times, were tested. IC_50_ values were calculated using a 4-parameter logistic curve fit. To investigate the time-dependent inhibition kinetics, compounds were pre-incubated with the described human USP18 enzyme at various time points (0, 0.5, 1, and 4 hours) before the addition of the substrate and at varying concentrations. An additional control was applied to the most promising hits in this study. An endpoint spike control was performed, in which USP18 reactions were run to the assay endpoint and quenched, as described. After that, compounds were added, and fluorescence was re-read to assess post-reaction quenching/inner-filter effects.

### Computational modeling.

AlphaFold 3 (48) was used to predict the structures of human USP18 and the USP18-ISG15 complex. Protein-ligand binding free energies were computed using the VeraChem Mining minima (VM2) method (49–53) utilizing the ff14SB (54) and GAFF2 (55) force fields for USP18 and ligand, respectively. For the VM2 computations, parameters were modified from default values, as follows: live/real = 8/6 Å; protein-ligand conformer search style = “vrigorous”; and protein-ligand number of search steps = 1,000.

Molecular dynamics (MD) simulations were performed using Amber 22 (56) with the ff19SB (57), GAFF2 (55), and 12-6 LJ nonbonded (58) force fields for protein, ligand, and Zn2^+^, respectively. Following VM2 protein-ligand minimizations, complexes were placed in a cuboid box with boundary of 12 Å, solvated with Optimal Point Charge (OPC) waters (59), neutralized with a Cl^–^, and additional Na^+^/Cl^–^ ions added to mimic salt concentration of 0.15M. MD runs were performed over 2 femtosecond time steps while constraining all hydrogen bonds via the Systematic Hard-Sphere Adjustment of Kinetic Energy (SHAKE) algorithm. A temperature of 300 K and pressure of 1atm in constant number of particles, pressure, and temperature (NPT) simulations were maintained using the Berendsen thermostat and Berendsen algorithm (60). A cutoff of 9 Å was applied to nonbonded interactions and explicit solvent periodic conditions were used.

For each simulation an initial multi-step equilibration process was performed starting with solvent treatment only (solute heavy atom restraint = 100): energy minimization, short period of heating from 100 K to 300 K (40 ps), and short MD at 300 K (50 ps). The complete system was then minimized and MD performed for 100 ps where both simulations used solute heavy atom constraint = 25. Next, several energy minimizations were performed with decreasing solute restraint (20, 15, 10, 5, 2.5, 1.0, 0.1, 0.01, and 0), before heating from 100 to 300 K (1 fs/Cα restraint = 10) followed by several MD runs at 300 K with varying times/restraints: 3 fs/Cα restraint = 1, 4 fs/Cα restraint = 0.1, 5 fs/no restraints. Lastly, production (unrestrained) MD runs were performed for 1 μs and these trajectories were analyzed using the cpptraj module.

Computation of the initial protein-ligand VM2 binding energy was started from a structure in which the ligand was placed in the hydrophobic pocket of USP18 above the F259 residue. Computation of the post-MD VM2 binding energy was started from the final MD structure (1 μs). PyMOL was used for mutagenesis and structure illustrations (61).

### Cell culture.

Human (H1299 and A549) and murine (344SQ) lung cancer cell lines were cultured in RPMI-1640 medium (#11875093, Thermo Fisher Scientific) supplemented with 10% fetal bovine serum (FBS, #A5256701, Thermo Fisher Scientific). Cells were cultured at 37°C in a humidified incubator with 5% CO_2_. The 344SQ murine lung cancer cell line was provided by Jonathan M. Kurie (MD Anderson Cancer Center). Human lung cancer cell lines H1299 and A549 were purchased and authenticated by American Type Culture Collection (ATCC).

### Small interfering RNAs and transfection.

Independent transient transfections of A549, H1299 and 344SQ lung cancer cells were with small interfering RNAs (siRNAs) following the manufacturer’s methods for siRNA-mediated transfection experiments (Dharmacon, Lafayette, CO) (29). Independent siRNAs to target USP18 and inactive control siRNAs were purchased (Dharmacon, Lafayette, CO); siRNAs that targeted USP18 were: USP18 human siRNA3 (5′-GGACUACCCUCAUGGCCUG-3′); and USP18 human siRNA4 (5′-GCAAAUCUGUCAGUCCAUC-3′). Transfection efficiency was measured by cotransfecting the siGLO Red Transfection Indicator (Dharmacon).

### Immunoblot assays.

Cells were lysed with ice-cold Pierce RIPA Lysis and Extraction Buffer (#89900, Thermo Fisher Scientific) supplemented with Halt Protease and Phosphatase Inhibitor Cocktail (#78440, Thermo Fisher Scientific). Proteins were resolved by SDS-PAGE before transfer to Trans-Blot Turbo Mini 0.2 μm PVDF Transfer Packs (#1704156, Bio-Rad). Membranes were blocked with 5% nonfat milk in Tris-buffered saline (#1706404XTU, Bio-Rad) and with 0.1% Tween-20 (#170653, Bio-Rad) (TBS-T) solution for at least 1 hour before overnight incubation at 4°C with a primary antibody diluted in 1% nonfat milk or 1% BSA in TBS-T. This was followed by 10-minute washes × 3 in TBS-T solution and with a 1-hour incubation with the desired secondary antibody diluted in 5% nonfat milk. After 3 additional washes, antibody binding was visualized by Clarity Western ECL Substrate (#1705061, Bio-Rad) and quantified by Image Lab software (Bio-Rad).

Anti-ISG15 antibody was provided by Shyamal Desai at the Department of Biochemistry and Molecular Biology, Louisiana State University Health Sciences Center School of Medicine (62). Primary antibodies were: Vinculin (#V4505, MilliporeSigma), GAPDH (#14C10, Cell Signaling Technology) and β-actin (#4967, Cell Signaling Technology). Secondary goat anti-mouse IgG (#1706515, Bio-Rad) and goat anti-rabbit IgG (#1706516, Bio-Rad) were used. Immunoblots were stripped using Restore PLUS Western Blot Stripping Buffer (#46430, Thermo Fisher Scientific) followed by TBS-T solution washing.

### Proliferation assays.

Logarithmically growing cells were seeded at optimized densities for each examined lung cancer cell line and cells were plated onto individual wells of 12-well tissue culture dishes, in triplicate. Cells were treated with β-lapachone at varying dosages and independently with the vehicle (DMSO) as a control 24 hours later. Proliferation was measured using the WST-1 assay (#5015944001, MilliporeSigma) after 24–72 hours of the indicated compound exposures on the FLUOstar Omega microplate reader (BMG Labtech). Proliferation studies were independently replicated at least 3 times.

### Apoptosis assays.

Logarithmically growing cells were seeded at optimized densities for each examined lung cancer cell line and cells were plated onto individual wells of 6-well tissue culture plates. Cells were treated with β-lapachone and independently with other candidate USP18 inhibitors at varying dosages and separately with vehicle (DMSO) 24 hours later. Apoptosis assays were performed using the FITC Annexin V Apoptosis Detection Kit (BioLegend) after 48 hours of drug exposure on a CytoFLEX flow cytometer (Beckman Coulter). Independent triplicate replicate experiments were performed.

### ROS assays.

ROS production was measured using the Total ROS Assay Kit 520 nm (#88-5930, Thermo Fisher Scientific). Briefly, cells were seeded at optimized densities into individual wells of 12-well tissue culture plates in triplicate, then treated with β-lapachone and independently with other USP18 candidate inhibitors at the indicated concentrations and durations as well as with the vehicle (DMSO) as a control. Cells were incubated with ROS assay stain solution 60 minutes prior to analysis on the CytoFLEX flow cytometer (Beckman). The ROS scavenger NAC (#A7250, MilliporeSigma) was used to interrogate ROS-dependent effects.

### IFN reporter assays.

Cells were seeded onto individual wells of 96-well tissue culture plates in triplicate 24 hours before transfection using the ISRE Reporter Kit (#60613, BPS Bioscience) and jetPRIME transfection reagent (#55-134, Genesee Scientific) following the manufacturer’s protocol. Cells were then treated with β-lapachone and independently with other candidate USP18 inhibitors at varying concentrations. Results were compared with vehicle controls. Firefly and Renilla luciferase signals were measured using the TWO-Step Luciferase Assay System (#60683, BPS Bioscience) and using the FLUOstar Omega microplate reader (BMG Labtech). The ratios of firefly luminescence signals to the corresponding Renilla luminescence signals were presented. The effects of cotransfection of wild-type as compared with mutant human USP18 species were explored.

### RNA-seq analysis.

The A549 lung cancer cell line was engineered with an insertless control vector or with a USP18 expression vector and transfectants were independently treated with vehicle (DMSO) or with β-lapachone at indicated dosages before harvesting tranfectants after 48 hours of these treatments. Comparisons were made to RNA-seq analysis performed in parental A549 lung cancer cells treated with β-lapachone at indicated dosages versus vehicle as a control. Total RNA was purified using the RNeasy Plus Universal Kit (#73404, Qiagen). The Illumina Stranded Total RNA library preparation ligation with Ribo-Zero Plus (#20040526, Illumina) was used for RNA-seq. Ribosomal RNA (rRNA) was removed using biotinylated target-specific oligos with the Ribo-Zero Plus rRNA Depletion Kit (#20037135, Illumina, Illumina).

RNA was fragmented and used to generate first strand cDNA using reverse transcriptase and random primers, followed by second strand cDNA synthesis using DNA Polymerase I and RNase H. The resulting double-strand cDNA was used as the input to a standard Illumina library preparation with end-repair, adapter ligation, and PCR amplification to yield a library for sequencing. The final purified product was quantitated by qPCR assays before cluster generation and sequencing on NextSeq 2000 P2 flowcell (Illumina, Illumina) for paired end 100-cycle sequencing.

The Illumina bcl2fastq (version 2.20) was used to demultiplex and convert binary base calls and qualities to the fastq format. The sequencing reads were trimmed to remove adapters and low-quality bases using Cutadapt (version 1.18). The trimmed reads were mapped to the human reference genome (hg38) and GENCODE annotation v30. The mapping was done using STAR aligner (version 2.7.0f) and with a 2-pass alignment option. RSEM (version 1.3.3) for gene and transcript quantification was based on the GENCODE M21 GTF file. To identify differentially expressed genes (DEGs), DESeq2 (version 1.44.0) was used. Volcano plots were generated using EnhancedVolcano (https://github.com/kevinblighe/EnhancedVolcano, commit ID 88a8e6a). Heatmap plots were generated using pheatmap (https://github.com/raivokolde/pheatmap). DEGs were scored using these criteria: adjusted *P* < 0.05 and fold change log_2_FC > 1. The statistically significant DEGs were analyzed using Metacore software (version 24.1, https://clarivate.com/products/metacore/) to perform pathway enrichment analyses and to generate pathway maps.

### qPCR assays.

qPCR assays were performed in triplicate, as previously described (63). The respective primers used were purchased from Thermo Fisher Scientific: human KLK2 primer (Hs00428384_g1); human IL31RA primer (Hs00371172_m1); human OTUD7A primer (Hs00370128_m1); human WASF3 primer (Hs00903488_m1); human NR4A2 primer (Hs01117527_g1); human DCC primer (Hs00180437_m1); human ATP6V1B1primer (Hs00266092_m1); human USP41primer (Hs02596851_gH); human PARP8 primer (Hs01065404_m1); human FGF13 primer (Hs00182807_m1); human ALDH1L1 primer (Hs01003842_m1); human ENTPD1 primer (Hs00969556_m1); human β-actin primer (Hs01060665_g1); and human USP18 primer (Hs00276441_m1).

### TCGA survival analysis.

Survival analyses were performed using the Kaplan-Meier Plotter (https://kmplot.com/analysis/), which integrates gene expression and clinical outcome data from TCGA pancancer cohort. For each indicated cancer type, patient datasets were analyzed independently within the corresponding TCGA cohort. Patients were stratified into high- and low-expression groups based on the median mRNA expression value of the gene of interest, applied as a predefined cutoff to avoid bias from data-driven or optimized threshold selection. Overall survival was evaluated using Kaplan-Meier estimates, and statistical significance was determined using the log-rank test.

### Lung cancer xenografts and drug treatments.

Human adenocarcinoma lung cancer H1299 cells (5 × 10^6^ cells) were injected s.c. into female NSG mice (Charles River Laboratories). These inbred mice were at least 8 weeks old and had at least a 1-week acclimation period before experiments began. Mice with palpable tumors were randomized based on similar basal tumor sizes to receive oral doses of vehicle, β-lapachone (25 mg/kg body weight; 5 days per week) or β-lapachone (12.5 mg/kg body weight; 5 days per week). There were 10 tumor-bearing mice in each arm. β-Lapachone was formulated in 2.5% DMSO/30% PEG400/67.5% Phosal MCT53 solution, and treatment results were compared with that of vehicle-treated mice. Body weights and tumor volumes were measured 3 times weekly. Tumor volumes were calculated as V = (length × width^2^)/2. H&E staining of lung cancers as well as Ki-67 staining for proliferation and the TUNEL assay for scoring of apoptosis were performed as previously described (64).

### Statistics.

Statistical analyses were done with the SPSS Statistics software (version 23, SPSS) and with GraphPad Prism software (version 8, GraphPad Software). Data were displayed as the mean and with Standard Deviations (SDs). Two-tailed Student’s *t* tests compared differences between study groups with a *P* value below 0.05 deemed statistically significant. Bonferroni corrections were used for multiple comparisons. Results of independent experiments were pooled to assess statistical significance. Experiments were typically performed in triplicate, with at least 3 independent biological replicate experiments.

### Study approval.

All methods were performed and reported in accordance with Animal Research: Reporting of In Vivo Experiments (ARRIVE) guidelines (https://arriveguidelines.org). Animal experiments were approved by the National Cancer Institute (NCI) Animal Care and Use Committee (ACUC).

### Data availability.

The datasets used as well as analyzed for this study will be available from the corresponding author upon reasonable request. The RNA seq data are available at the Gene Expression Omnibus accession GSE318135 (https://www.ncbi.nlm.nih.gov/geo/query/acc.cgi?acc=GSE318135). Values for all data points in graphs are reported in the Supporting Data Values file.

## Author contributions

ED and Xi Liu conceived the study design, supervised the study, and interpreted the data. SDD and GGP provided key reagents. KND, BOO, SCO, EJC, BL, and GGP interpreted the data. BOO, SCO, EJC, LTM, ZC, Xiuxia Liu, BL, YA, AKD, and RMS collected data. JI and BL performed modeling studies. Xi Liu, BOO, KND, and ED wrote this manuscript.

## Conflict of interest

The authors have declared that no conflict of interest exists.

## Funding support

This work is the result of NIH funding, in whole or in part, and is subject to the NIH Public Access Policy. Through acceptance of this federal funding, the NIH has been given a right to make the work publicly available in PubMed Central.

- NCI Intramural Research Program and Center for Cancer Research, NCI.
- NIH contract no. 75N91019D00024 (ED).
- Frederick National Laboratory Research Support Program (XL and BL).
- Grant from the Samuel Waxman Cancer Research Foundation (ED).
- American Cancer Society Professorship (ED).
- The Cancer Prevention and Research Institute of Texas (CPRIT) grant RP210088 (KND).

## Supplementary Material

Supplemental data

Unedited blot and gel images

Supporting data values

## Version 1 05/19/2026

In-Press Preview

## Version 2 07/22/2026

Electronic publication
