# Supplementary material for: Derivation and characterization of ubiquitin-specific protease 18 inhibitors
Source: JCI Insight. 2026 May 19;11(14):e202853. doi: 10.1172/jci.insight.202853 (PMC13461152; doi:10.1172/jci.insight.202853)
Supplement: Supplementary file 1 [file jciinsight-11-202853-s203.pdf]

Supplementary Fig. 1

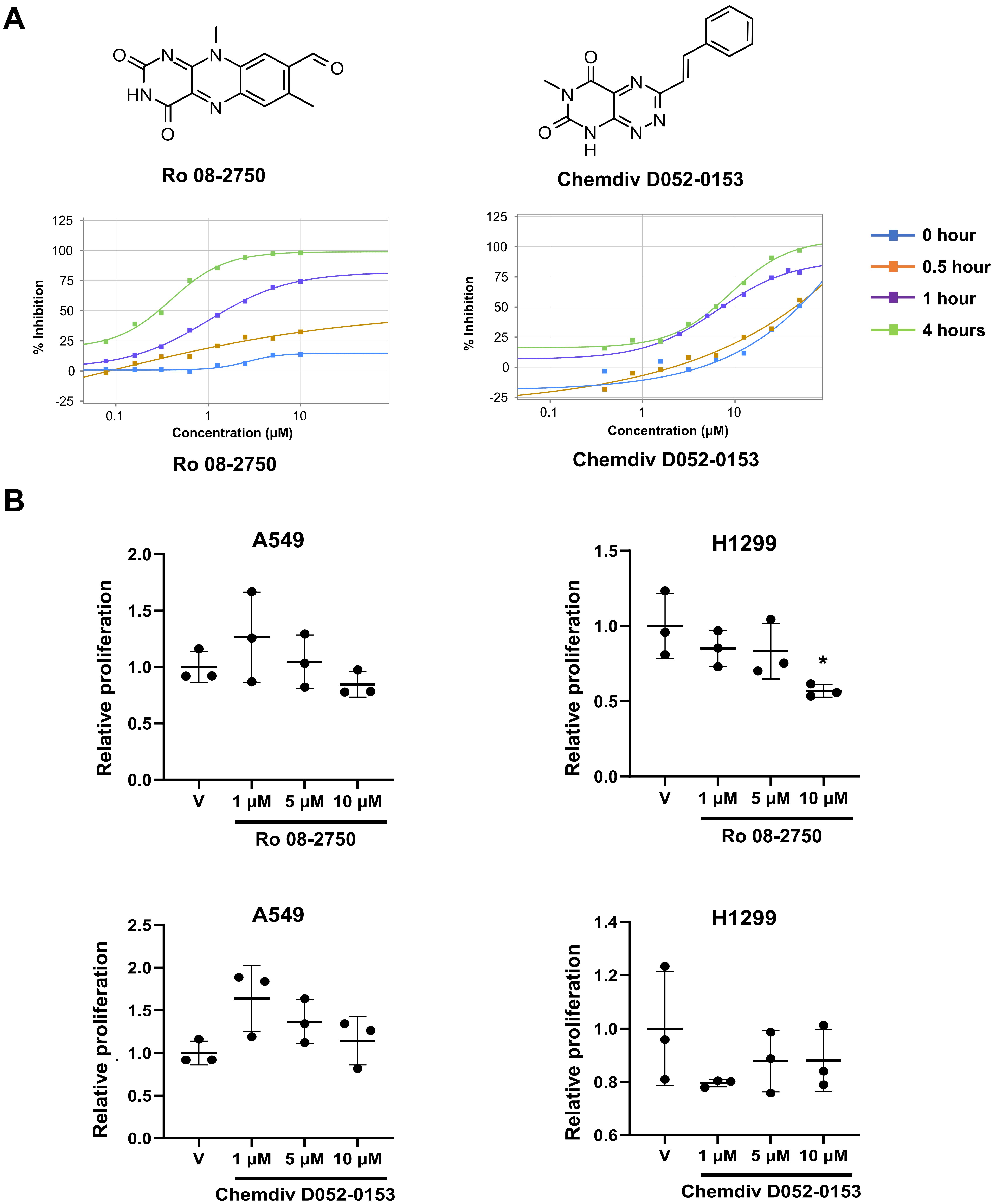

Supplementary Figure 1: Dose–response and proliferative effects of additional USP18-inhibitory compounds. (A) Chemical structures and in vitro dose–response inhibition curves of Ro 08-2750 and ChemDiv D052-0153 compounds in the USP18 enzymatic assay. Enzymatic activity was measured at multiple time points (0, 0.5, 1, and 4 hours) after compound addition to assess time-dependent inhibition. (B) Effects of Ro 08-2750 and ChemDiv D052-0153 on proliferation independently in A549 and H1299 human lung cancer cells following 48-hour treatment at the indicated concentrations. V is vehicle. Two-tailed Student's t tests compared differences between treatment arms with a P-value < 0.05 deemed statistically significant. Error bars represent standard deviations and with the symbol indicating * P < 0.05.

Supplementary Fig. 2

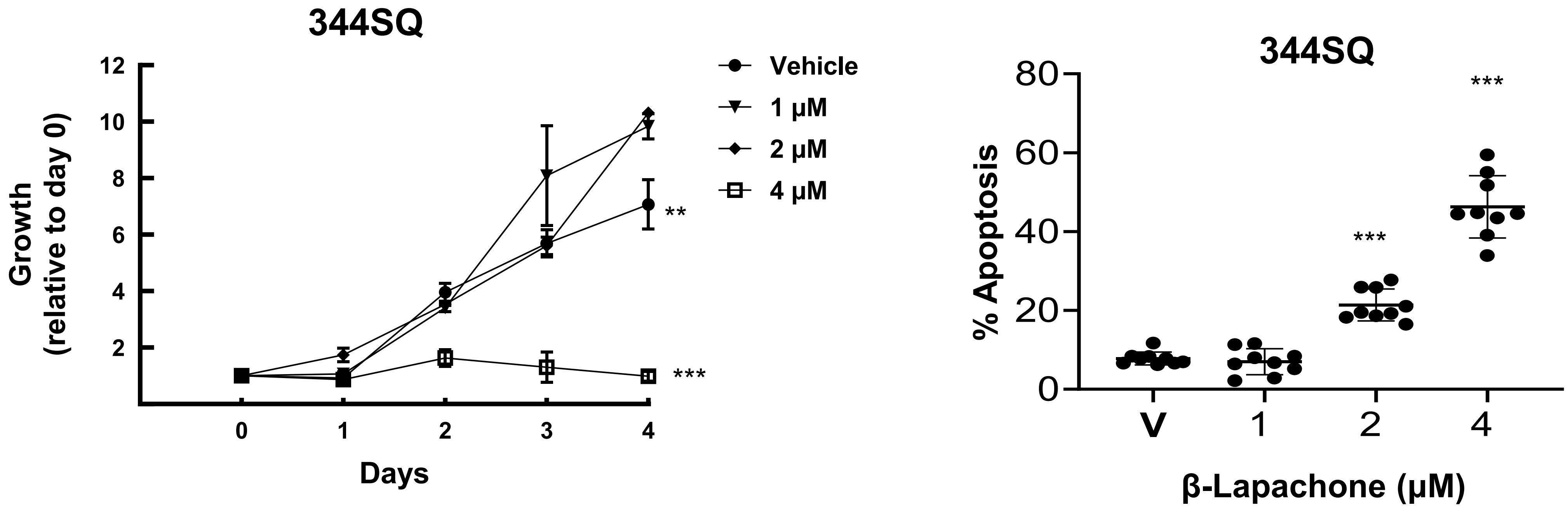

Supplementary Figure 2: β-lapachone treatment reduces proliferation and augments apoptosis in murine lung cancer cells. Proliferation (left) and apoptosis (right) assays were performed in 344SQ murine lung cancer cells treated with increasing concentrations of β-lapachone for 48 hours. β-lapachone statistically significantly reduced cell proliferation and increased apoptosis in a dose-dependent manner. V is vehicle. Two-tailed Student's t tests compared differences between arms and with a P-value < 0.05 deemed statistically significant. Error bars represent standard deviations and with these symbols indicating ** P < 0.01 and *** P < 0.001, respectively.

Supplementary Fig. 3

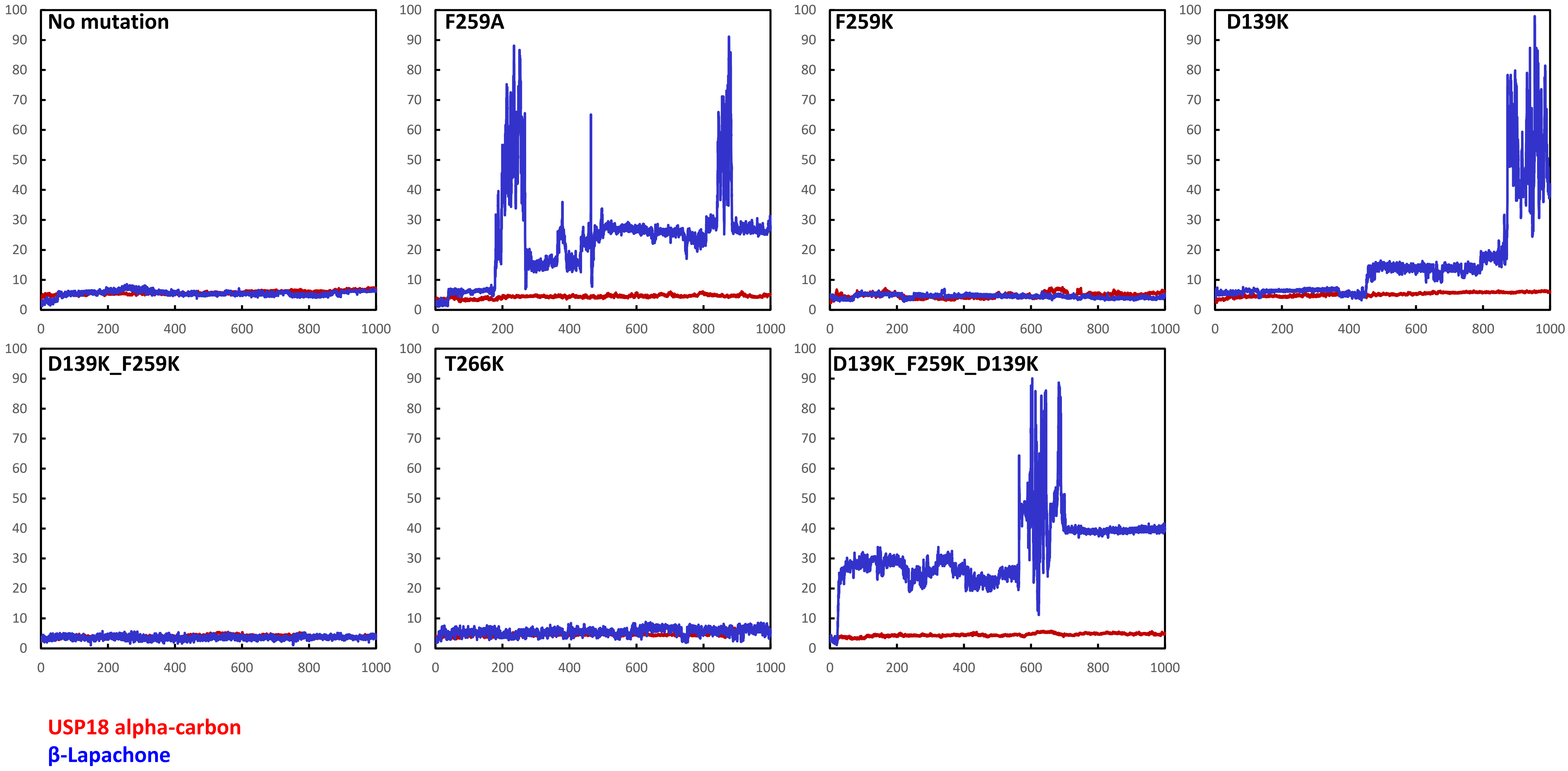

Supplementary Figure 3: Molecular dynamics simulation of β-lapachone binding to wild-type as compared to mutant USP18 proteins. Root mean-square deviation (RMSD), from the starting structure, over a 1000 ns molecular dynamics simulation are shown for wild-type and independently for mutant USP18 proteins complexed with β-lapachone. The x-axis represents time (ns) while the y-axis represents RMSD from the starting structure. Each panel represents a distinct USP18 variant, with red signals depicting the USP18 α-carbon backbone and blue signals representing β-lapachone. The trajectories illustrate the stability and interaction dynamics of β-lapachone binding to each USP18 variant over time.

Supplementary Fig. 4

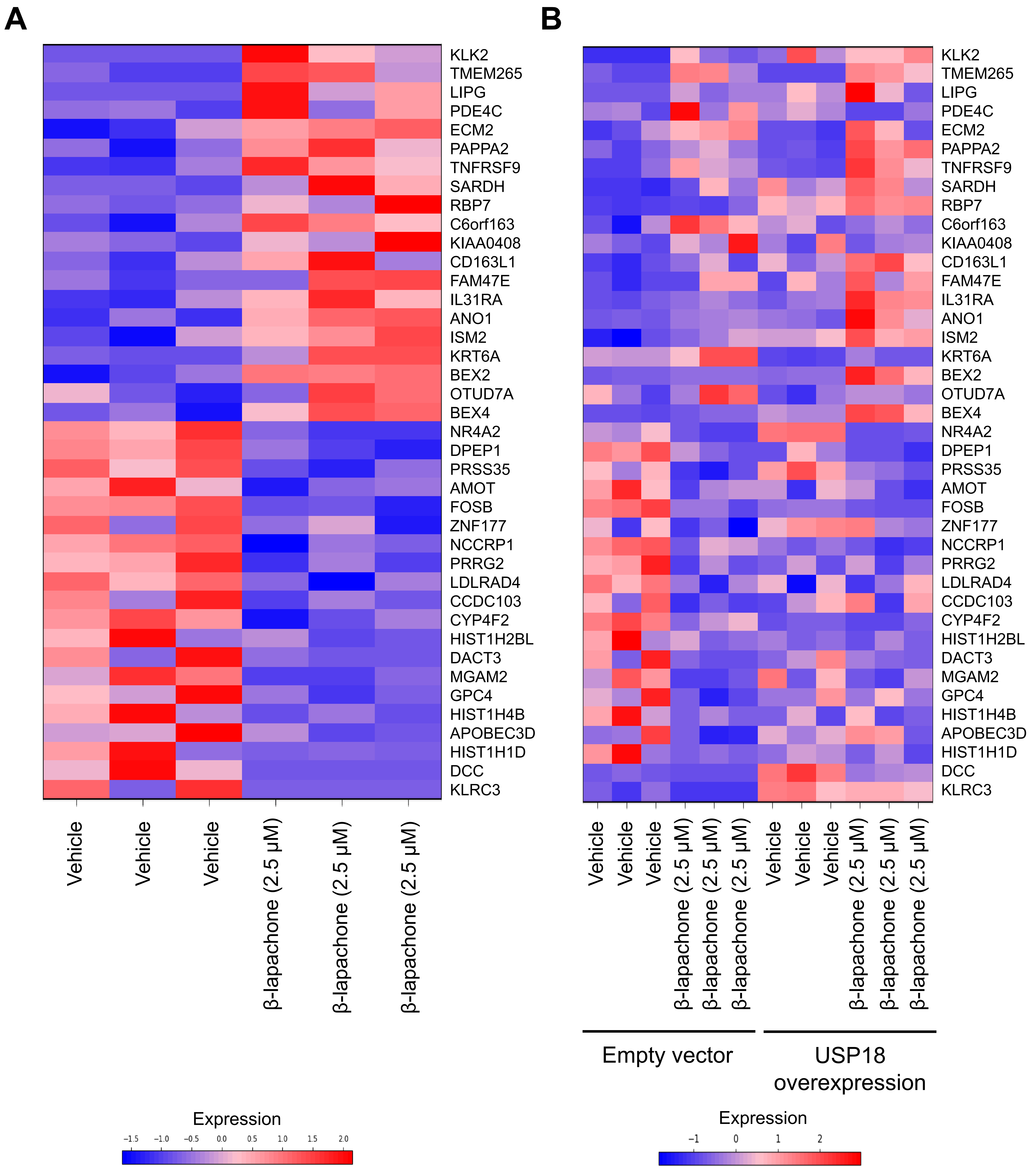

Supplementary Fig. 4 continued

C

Up-Regulated Expression		
Gene List	Fold change	P-value
KLK2	24.44	0.0007
TMEM265	16.67	0.0206
LIPG	13.19	0.0105
FAM225A	11.00	0.0154
PDE4C	4.64	0.0217
ECM2	4.46	0.0280
ANKRD20A3	3.94	0.0126
COX6A1P2	3.81	0.0141
PAPPA2	3.80	0.0134
TNFRSF9	3.54	0.0000
SARDH	3.51	0.0097
RBP7	3.24	0.0066
C6orf163	3.16	0.0201
C7orf65	3.06	0.0341
AACSP1	2.69	0.0247
KIAA0408	2.68	0.0469
CD163L1	2.64	0.0104
FAM47E	2.60	0.0360
IL31RA	2.60	0.0006
ANO1	2.43	0.0094
ISM2	2.36	0.0111
KRT6A	2.35	0.0124
WDR66	2.33	0.0157
AMT	2.31	0.0217
BEST3	2.20	0.0361
APLN	2.17	0.0015
BEX2	2.11	0.0000
PLA2G4C	2.10	0.0002
KCNG3	2.10	0.0002
PLEK2	2.07	0.0000
PLTP	2.04	0.0001
AZIN2	2.02	0.0005
OTUD7A	2.01	0.0441
BEX4	2.01	0.0032

Down-Regulated Expression		
Gene List	Fold change	P-value
SPTLC3	-2.00	0.0063
CRYAB	-2.08	0.0053
GUCY2EP	-2.14	0.0500
TMEM105	-2.15	0.0007
WASF3	-2.17	0.0106
CPLX1	-2.18	0.0060
SNCA	-2.19	0.0107
GUCY1B1	-2.22	0.0064
ATP6V1B1	-2.22	0.0006
NR4A2	-2.24	0.0000
DPEP1	-2.27	0.0060
LINC00319	-2.28	0.0056
PRSS35	-2.45	0.0009
LINC01275	-2.45	0.0161
AMOT	-2.46	0.0166
FOSB	-2.47	0.0000
ZNF177	-2.49	0.0043
NCCRP1	-2.51	0.0347
PRRG2	-2.56	0.0063
LDLRAD4	-2.62	0.0477
CCDC103	-2.69	0.0372
RN7SL2	-2.72	0.0111
CYP4F2	-2.80	0.0086
HIST1H2BL	-2.86	0.0245
DACT3	-2.92	0.0032
RN7SL1	-2.99	0.0044
MGAM2	-3.25	0.0322
RN7SL3	-3.37	0.0112
GPC4	-3.64	0.0076
SNORA73B	-4.51	0.0083
HIST1H4B	-5.30	0.0207
APOBEC3D	-5.45	0.0037
RF00003	-7.11	0.0173
HIST1H1D	-7.17	0.0012
DCC	-8.88	0.0234
KLRC3	-12.02	0.0097
RPPH1	-14.30	0.0001
CSPG4P5	-15.31	0.0408

D **Lung squamous cell carcinoma**

USP18

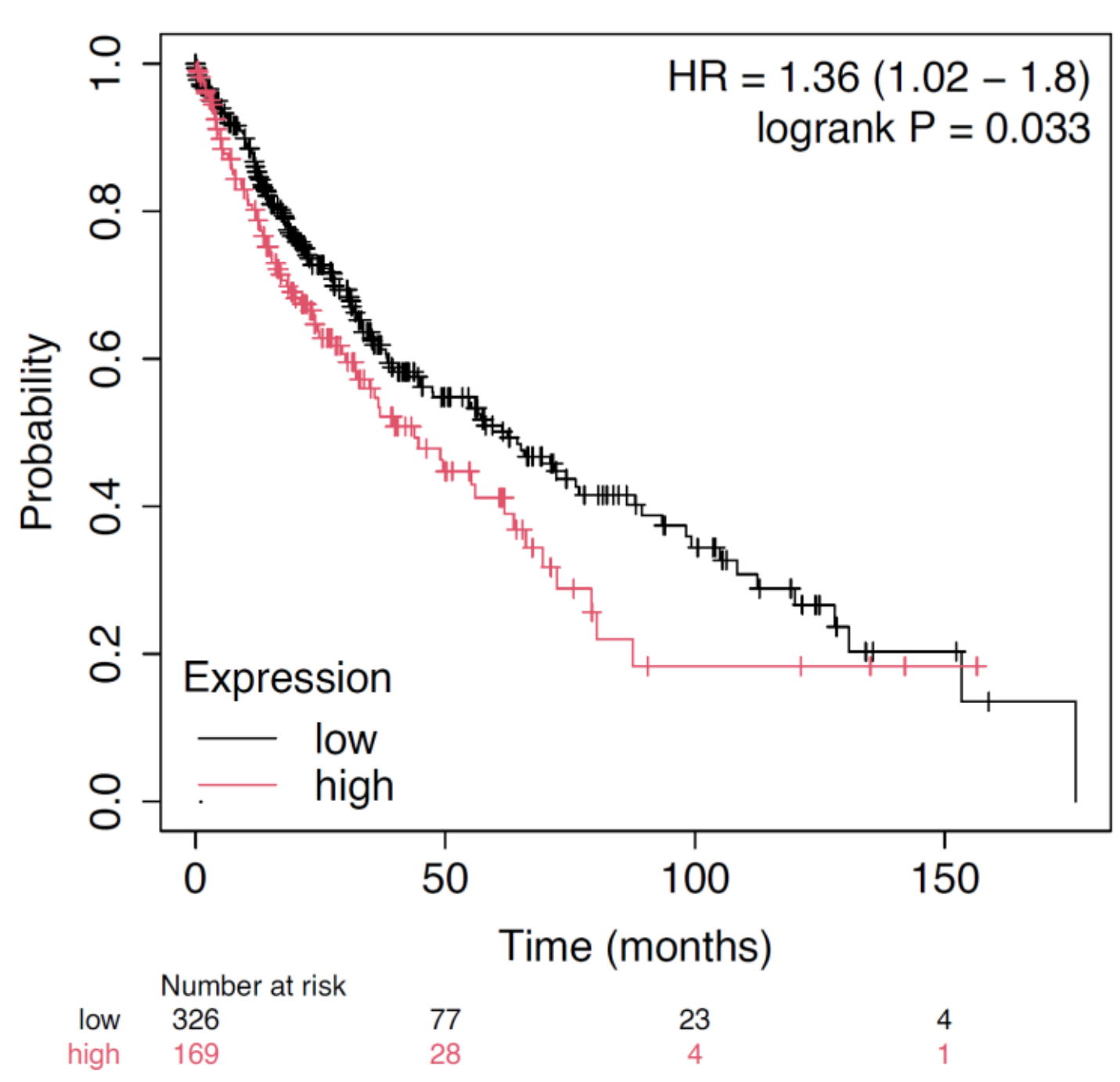

ISG15

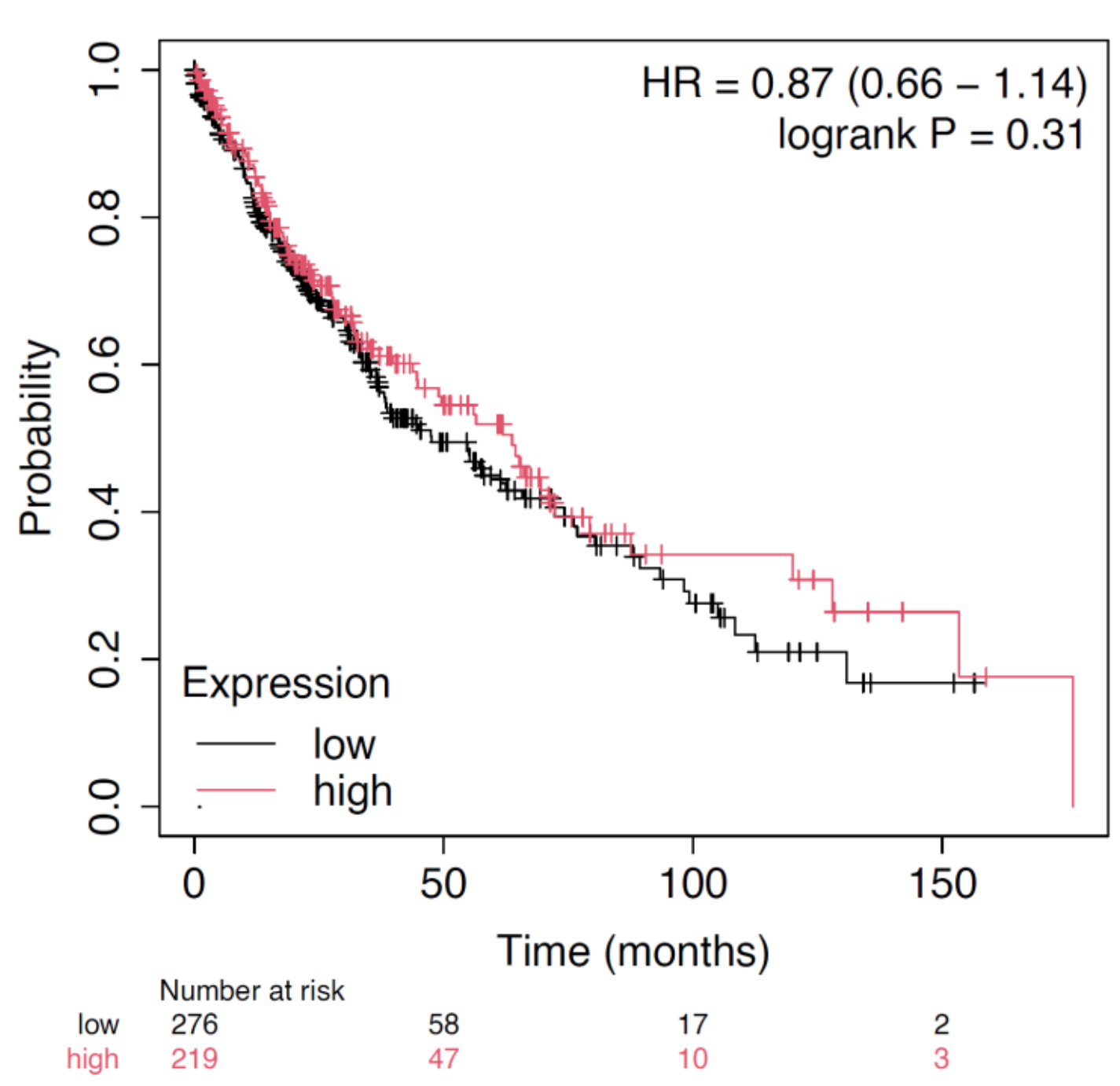

Supplementary Figure 4: Transcriptomic changes and survival associations of expressed USP18–ISG15 pathway genes in lung cancers. (A) Heatmap plot displayed the differentially expressed genes in A549 human lung cancer cells treated with β-lapachone (2.5 μM) as compared to vehicle-treated controls. (B) Heatmap plot of A549 lung cancer cells engineered to over-express USP18 versus empty vector controls is shown following β-lapachone treatment (2.5 μM) as compared to vehicle control. (C) Differentially expressed genes regulated by β-lapachone treatment that were previously linked to the USP18–ISG15 pathway. Up-regulated and down-regulated transcripts are displayed. (D) Kaplan–Meier survival analyses of The Cancer Genome Atlas (TCGA) for the lung squamous cell carcinoma cohort. The independent effects of USP18 or ISG15 mRNA expression on overall survival of lung squamous cell carcinomas are shown.

Supplementary Fig. 5

Up-Regulated Protein Expression

Protein Name	Protein Symbol
Putative annexin A2-like protein	ANXA2P2
CREB-binding protein	CREBBP
Putative ubiquitin-conjugating enzyme E2 N-like	UBE2NL
Dynein heavy chain 8, axonemal	DNAH8
Dynein light chain 1, cytoplasmic	DYNLL1
Putative 60S ribosomal protein L13a protein RPL13AP3	RPL13AP3
Transcription factor HIVEP2	HIVEP2
Unconventional myosin-VIIa	MYO7A
Coiled-coil domain-containing protein 66	CCDC66
Eukaryotic translation initiation factor 5A-1-like	EIF5A1P3
Zinc finger protein ZFPM2	ZFPM2
Fructose-bisphosphate aldolase C	ALDOC
Hemoglobin subunit delta	HBD
MAP7 domain-containing protein 1	MAP7D1
Band 4.1-like protein 1	EPB41L1
cAMP-dependent protein kinase type I-alpha regulatory subunit	PRKAR1A
Laminin subunit beta-3	LAMB3
Barrier-to-autointegration factor	BANF1
Coactosin-like protein	COTL1
Cytochrome P450 2A13	CYP2A13
Envoplakin	EVPL
Geranylgeranyl transferase type-2 subunit alpha	RABGGTA
Myosin-binding protein H	MYBPH
Small integral membrane protein 32	SMIM32
Solute carrier family 15 member 4	SLC15A4
Steroidogenic acute regulatory protein, mitochondrial	STAR
Uncharacterized protein C1orf185	C1orf185
WD repeat-containing protein 17	WDR17

Down-Regulated Protein Expression

Protein Name	Protein Symbol
Methylmalonic aciduria and homocystinuria type D protein, mitochondrial	MMADHC
Kinesin-like protein KIF26B	KIF26B
Structural maintenance of chromosomes protein 4	SMC4
Complement C3	C3
STE20-like serine/threonine-protein kinase	SLK
Complement C4-A	C4A
Golgi-specific brefeldin A-resistance guanine nucleotide exchange factor 1	GBF1
Probable non-functional immunoglobulin heavy variable 3-38	IGHV3-38
Aldo-keto reductase family 1 member C1	AKR1C1
Titin	TTN
HAUS augmin-like complex subunit 6	HAUS6
ATP-binding cassette sub-family A member 5	ABCA5
Carbonic anhydrase 6	CA6
Centromere protein Q	CENPQ
Mitochondrial 2-oxoglutarate/malate carrier protein	SLC25A11
Putative olfactory receptor 1F2	OR1F2P
CMRF35-like molecule 5	CD300LD
RNA cytosine C(5)-methyltransferase NSUN2	NSUN2
Histone H3.2	HIST2H3A
Heterogeneous nuclear ribonucleoprotein L-like	HNRNPLL
Late cornified envelope protein 1A	LCE1A
Zinc finger protein 236	ZNF236
Carbohydrate sulfotransferase 4	CHST4
Histone H2A type 2-C	HIST2H2AC
Histone-lysine N-methyltransferase KMT5B	KMT5B
Pro-neuregulin-2, membrane-bound isoform	NRG2
Transcriptional adapter 2-beta	ADA2B

Supplementary Figure 5: Proteomic profiling of β-lapachone–regulated ISG15-conjugated proteins. Proteomic analysis was performed under stringent immunoprecipitation conditions to identify candidate covalently ISG15-conjugated proteins following β-lapachone treatment. The up-regulated (left) and down-regulated (right) ISG15-conjugated proteins are shown, respectively. These results identify candidate ISG15 substrates modulated by USP18 inhibition following β-lapachone versus vehicle treatments.

Supplementary Fig. 6

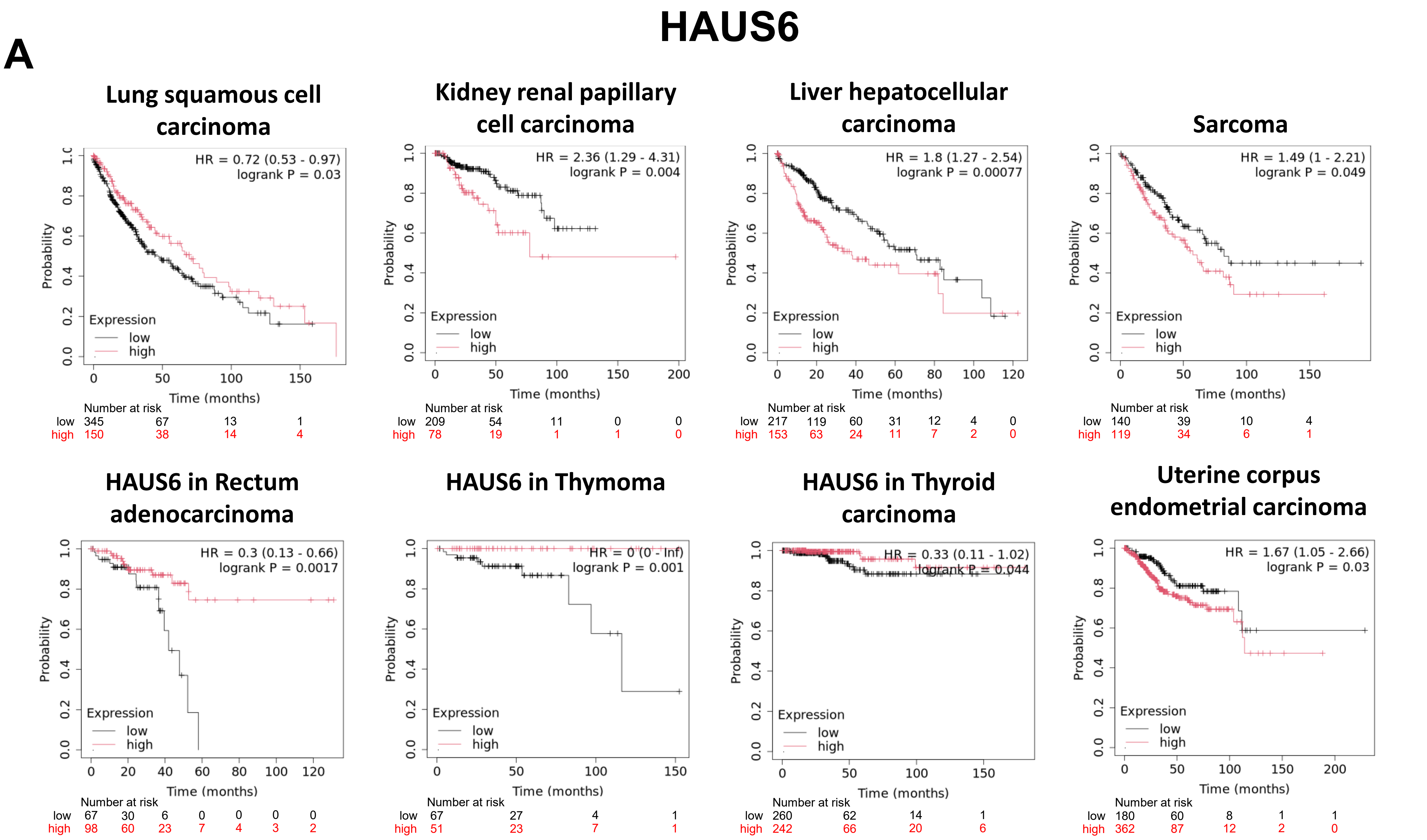

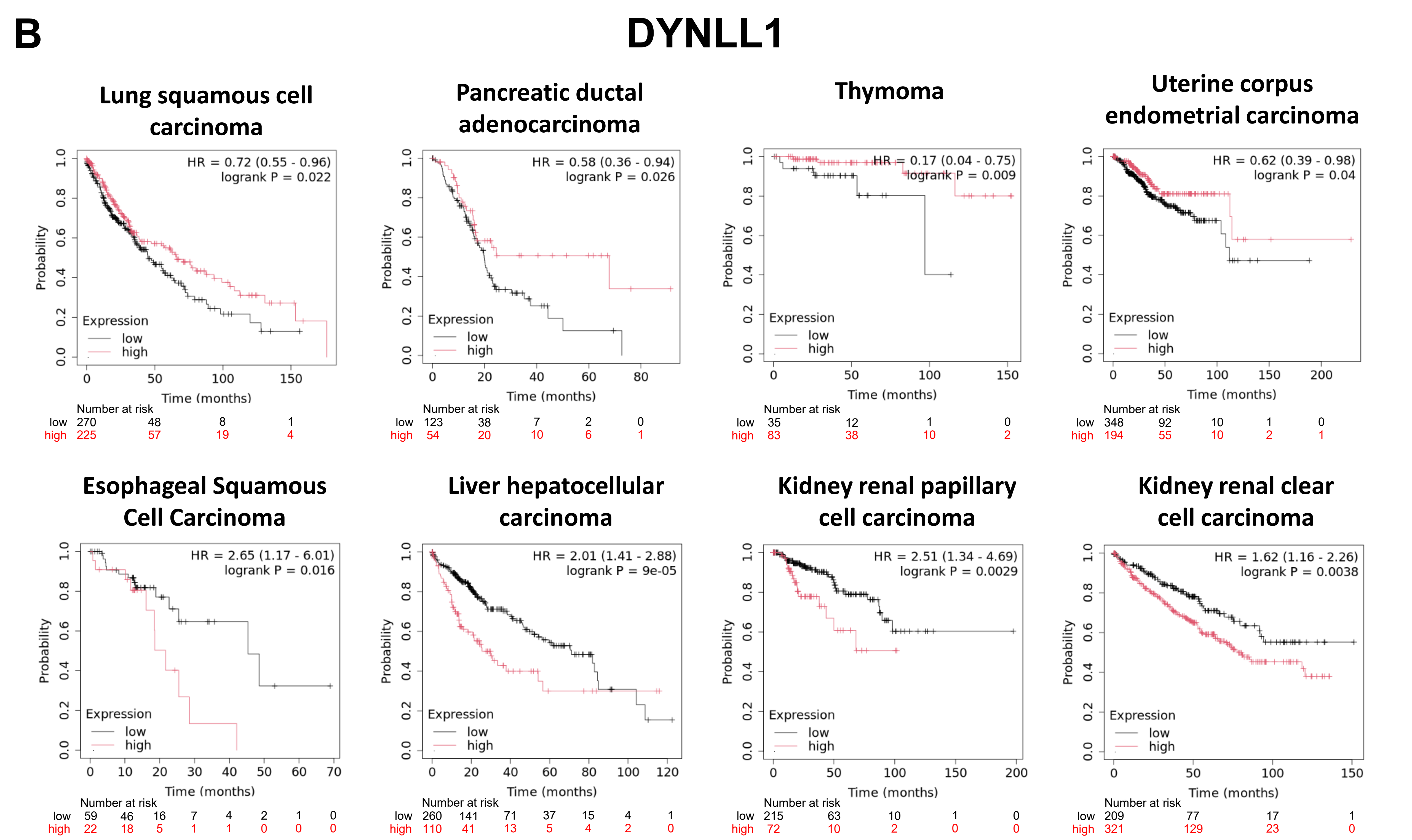

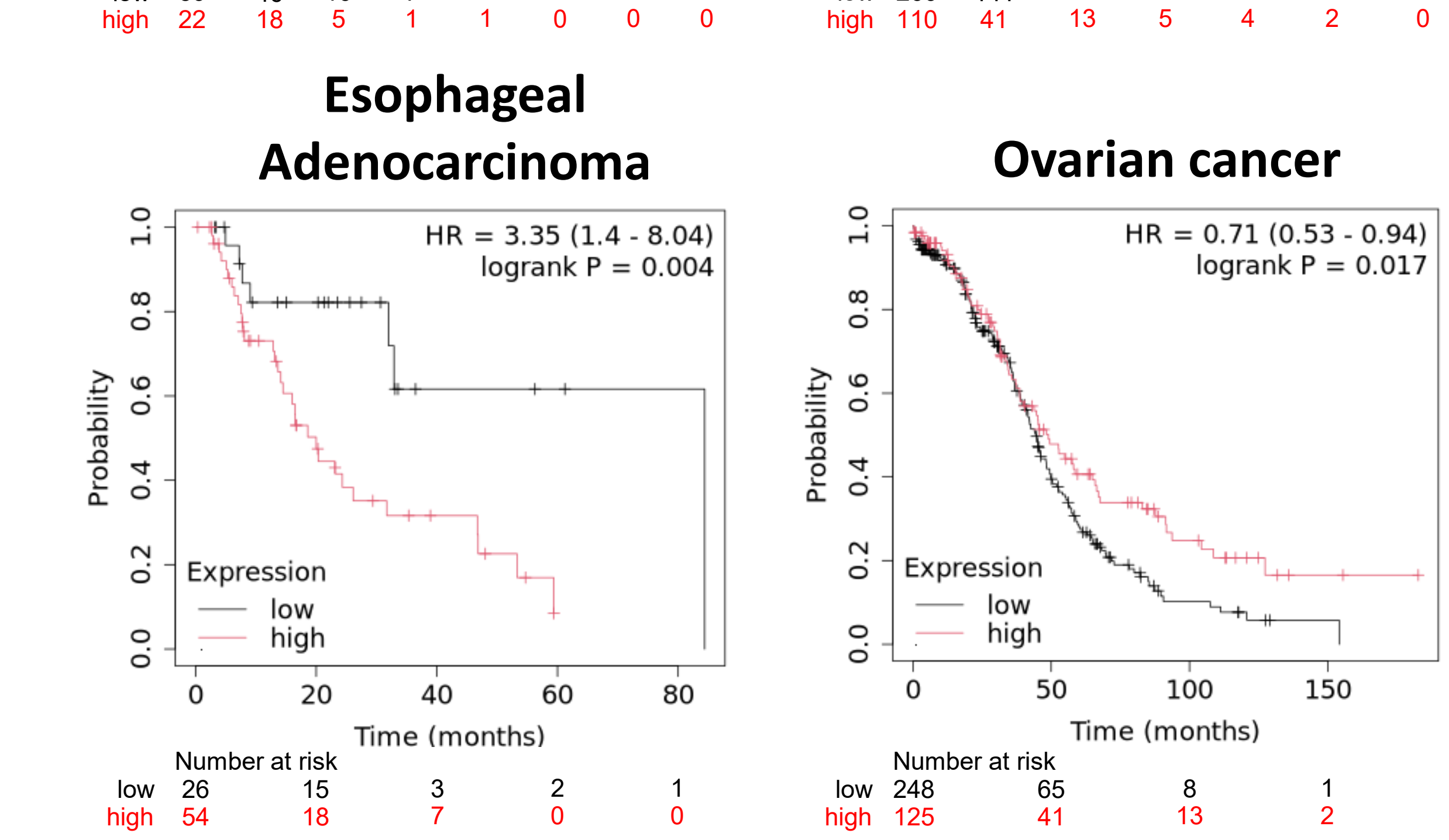

Supplementary Figure 6: Independent TCGA survival analyses for differing mRNA expression levels of (A) HAUS6 and (B) DYNLL1. These species were identified as ISG15-conjugated proteins in the proteomics analysis and are significantly associated with patient survival in multiple cancer types, as shown here.
