# Supplementary material for: Derivation and characterization of ubiquitin-specific protease 18 inhibitors
Source: JCI Insight. 2026 May 19;11(14):e202853. doi: 10.1172/jci.insight.202853 (PMC13461152; doi:10.1172/jci.insight.202853)
Supplement: Supplementary file 2 [file jciinsight-11-202853-s204.pdf]

Figure 1

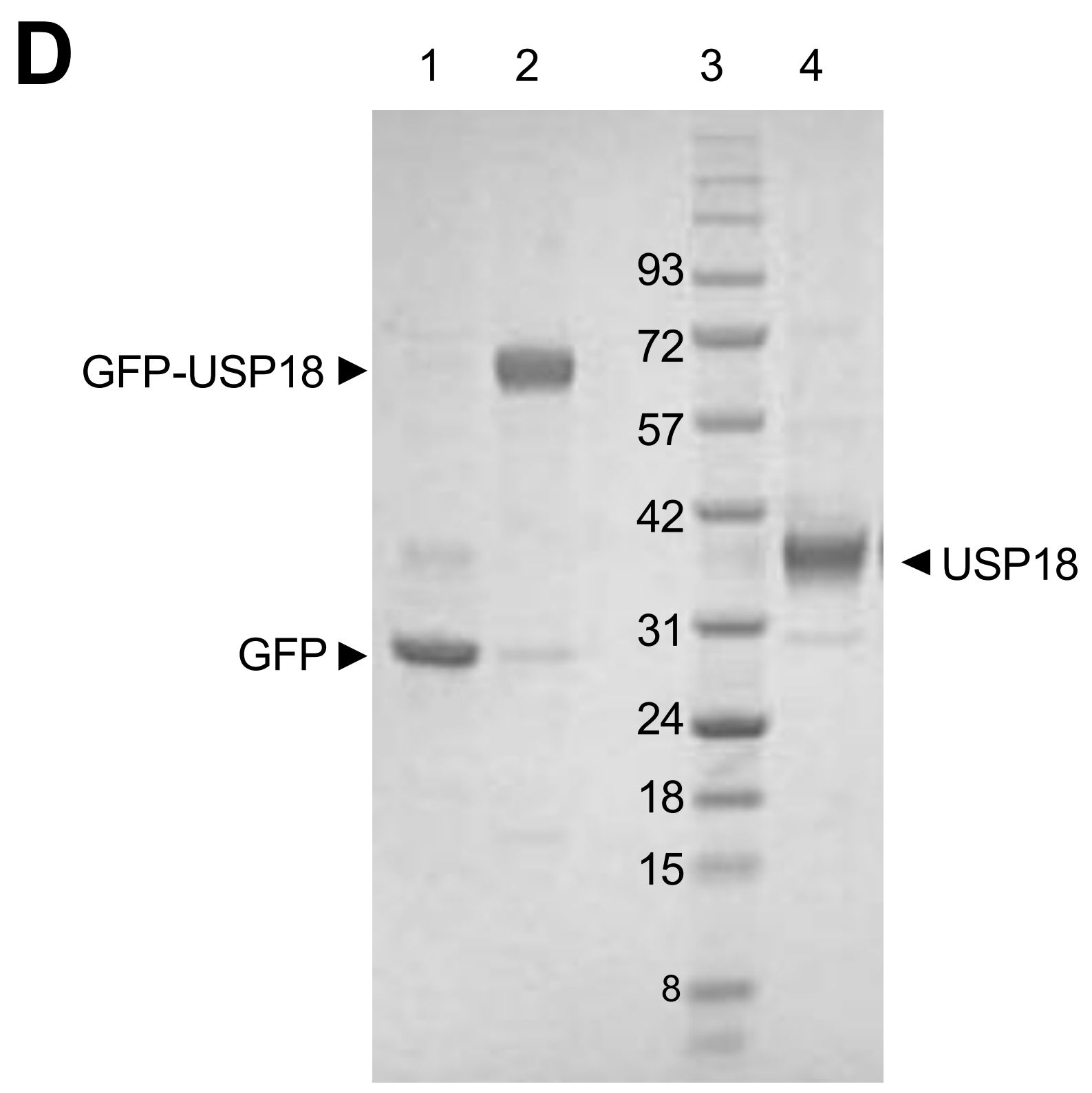

Figure 3B

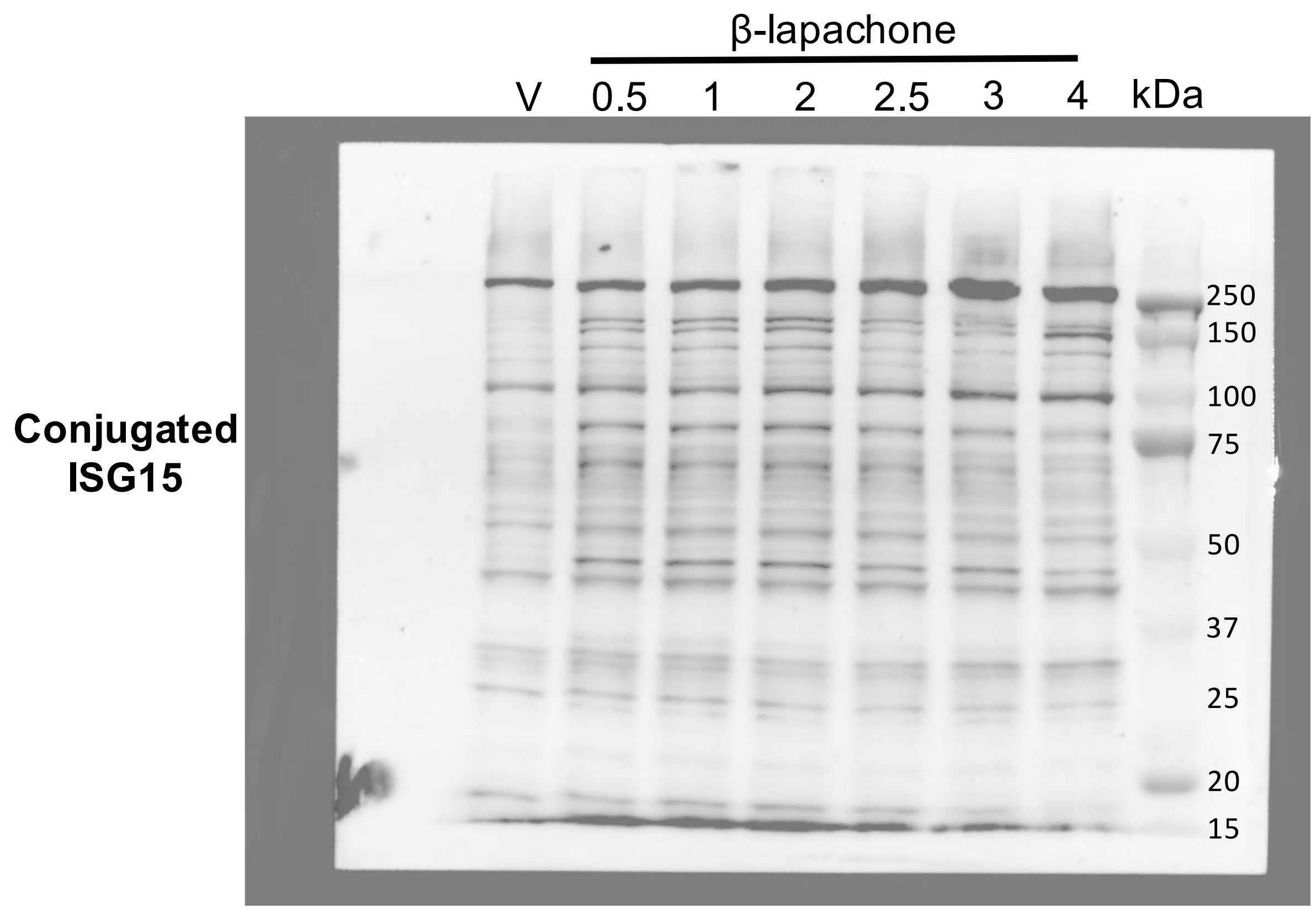
β-lapachone
V 0.5 1 2 2.5 3 4 kDa
Conjugated ISG15
250
150
100
75
50
37
25
20
15

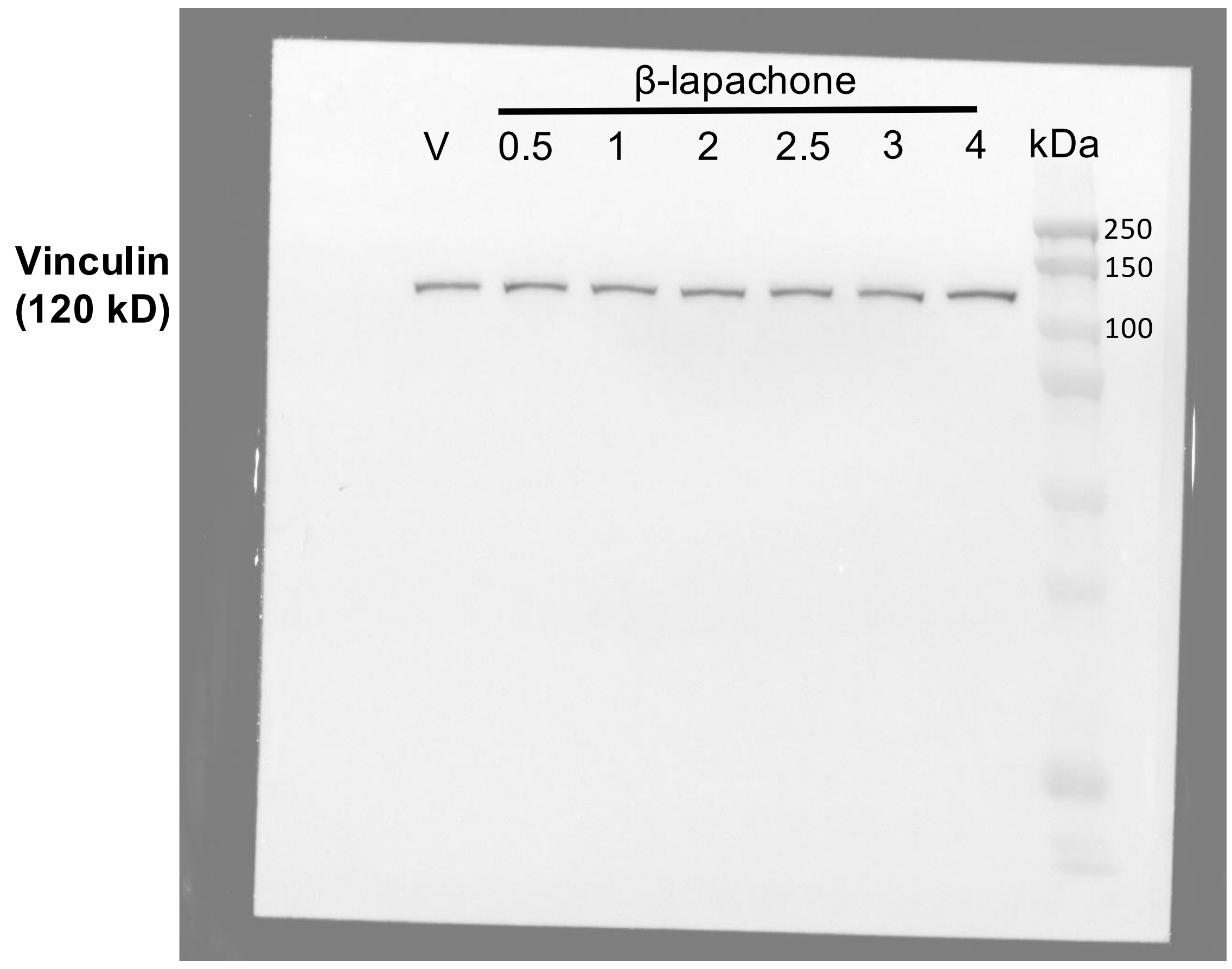
β-lapachone
V 0.5 1 2 2.5 3 4 kDa
Vinculin (120 kD)
250
150
100

Figure 3C

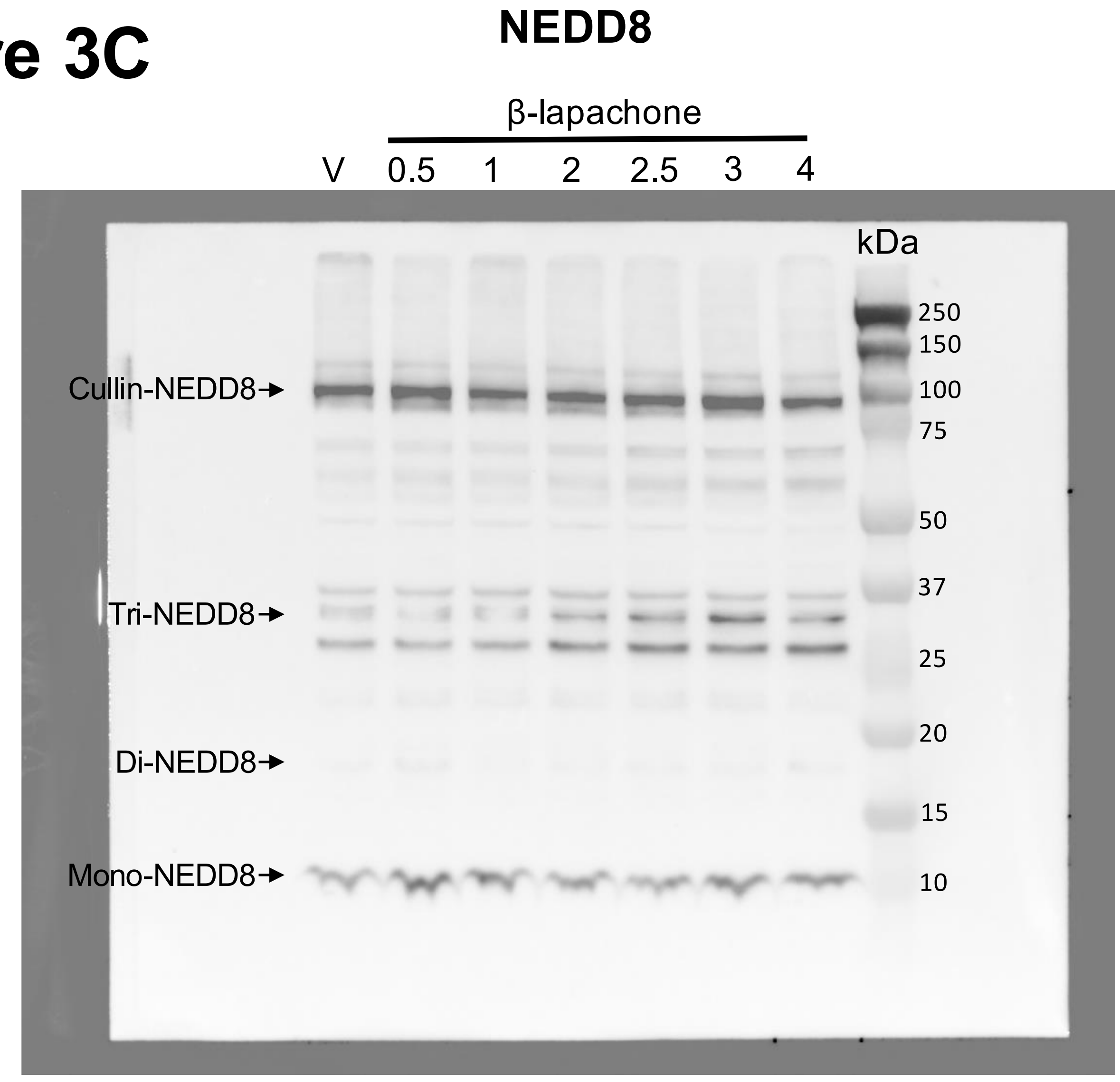

NEDD8
β-lapachone
V
0.5
1
2
2.5
3
4
kDa
250
150
100
75
50
37
25
20
15
10
Cullin-NEDD8
Tri-NEDD8
Di-NEDD8
Mono-NEDD8

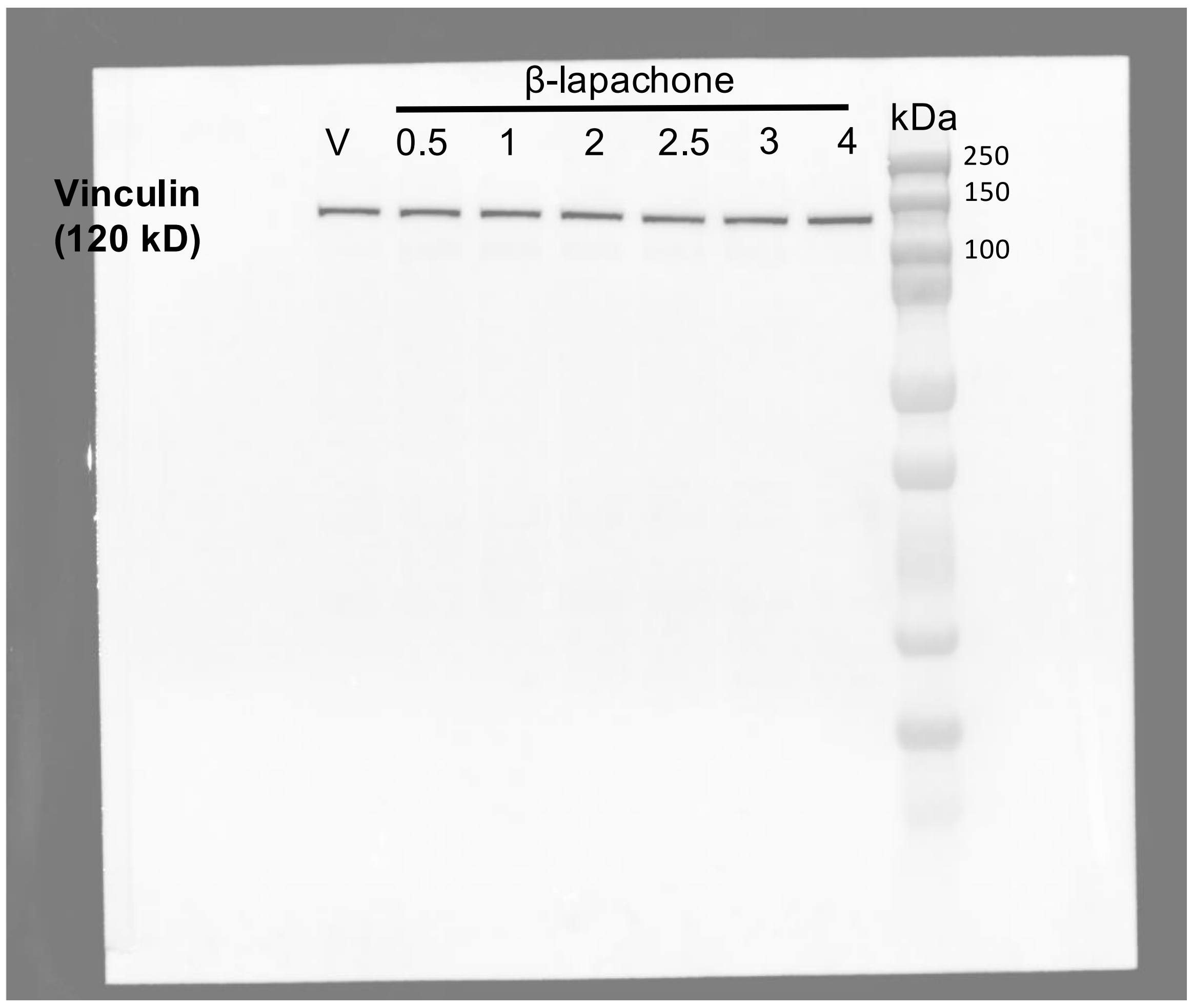

β-lapachone
V
0.5
1
2
2.5
3
4
kDa
250
150
100
Vinculin
(120 kD)

Figure 3C

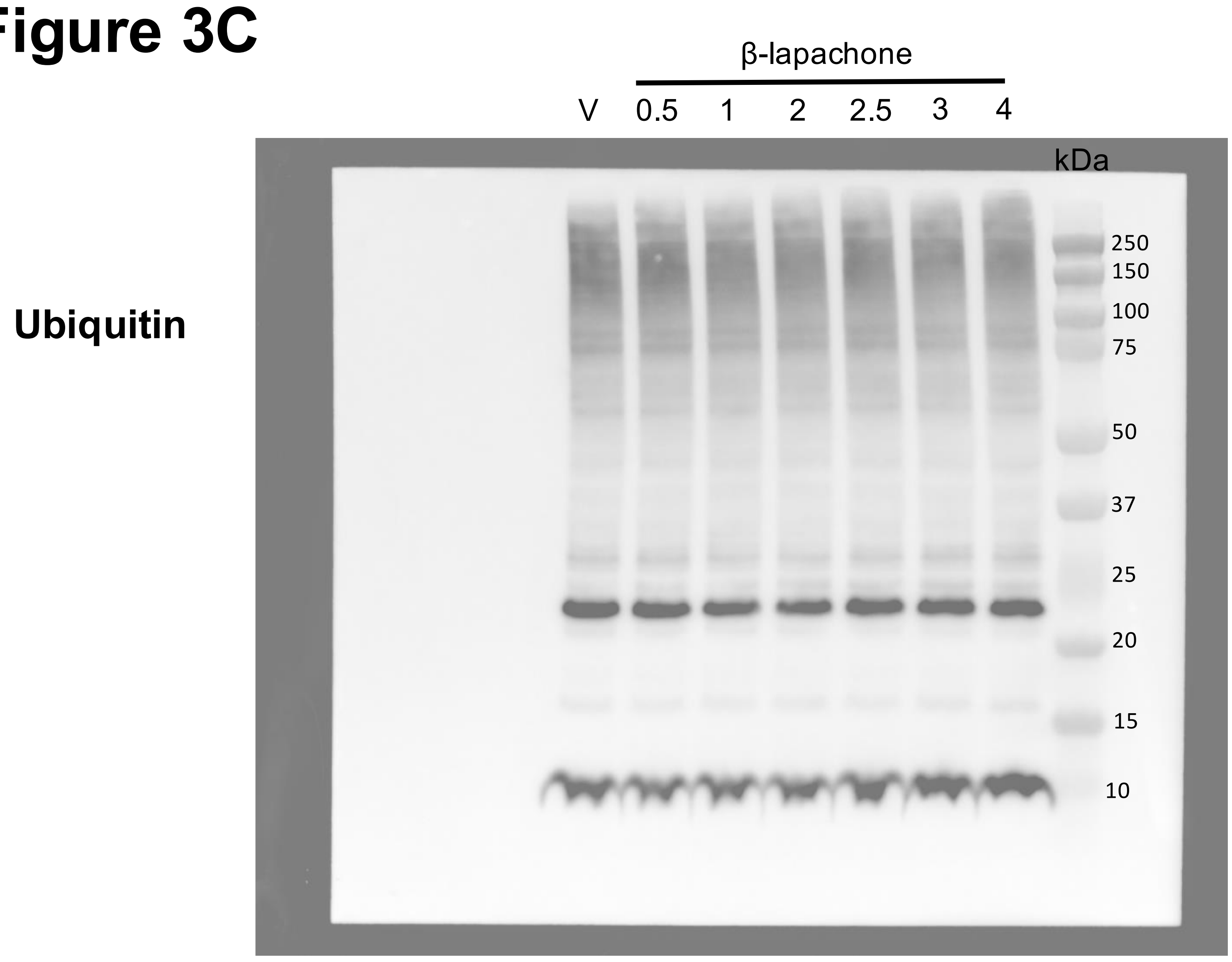

Ubiquitin
β-lapachone
V 0.5 1 2 2.5 3 4
kDa
250
150
100
75
50
37
25
20
15
10

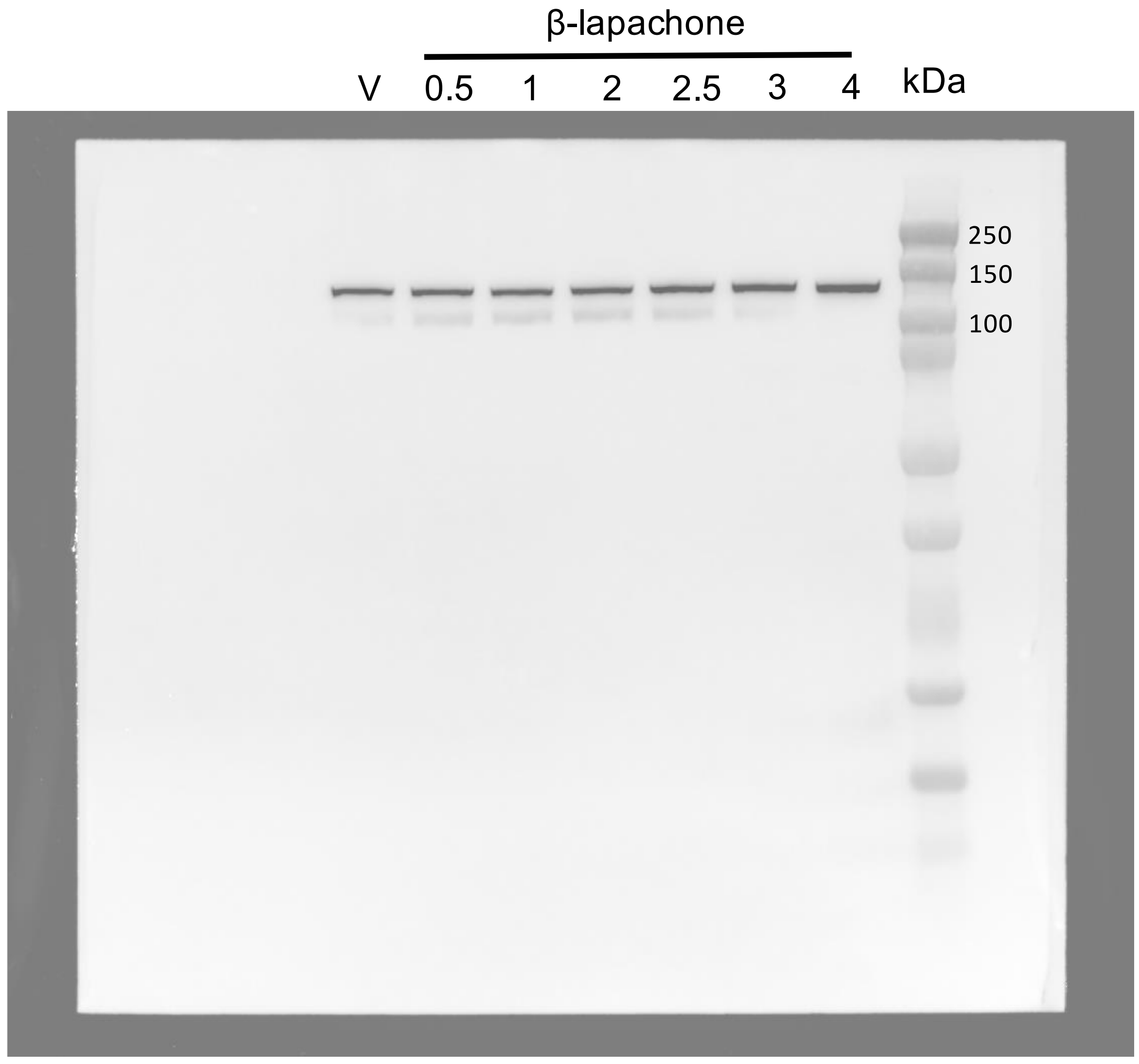

β-lapachone
V 0.5 1 2 2.5 3 4
kDa
250
150
100

Figure 3C

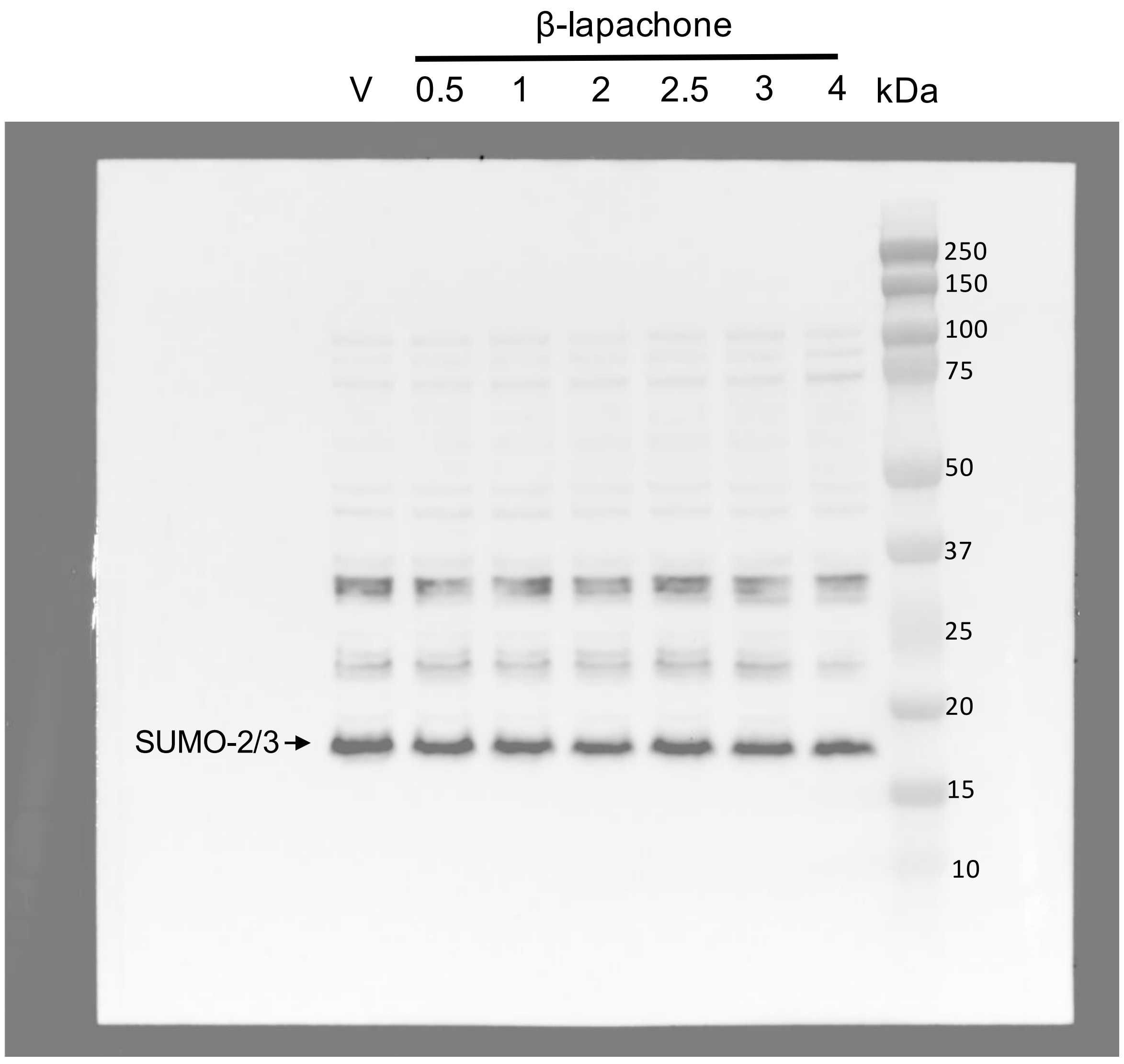
β-lapachone
V 0.5 1 2 2.5 3 4 kDa
250
150
100
75
50
37
25
20
SUMO-2/3
15
10

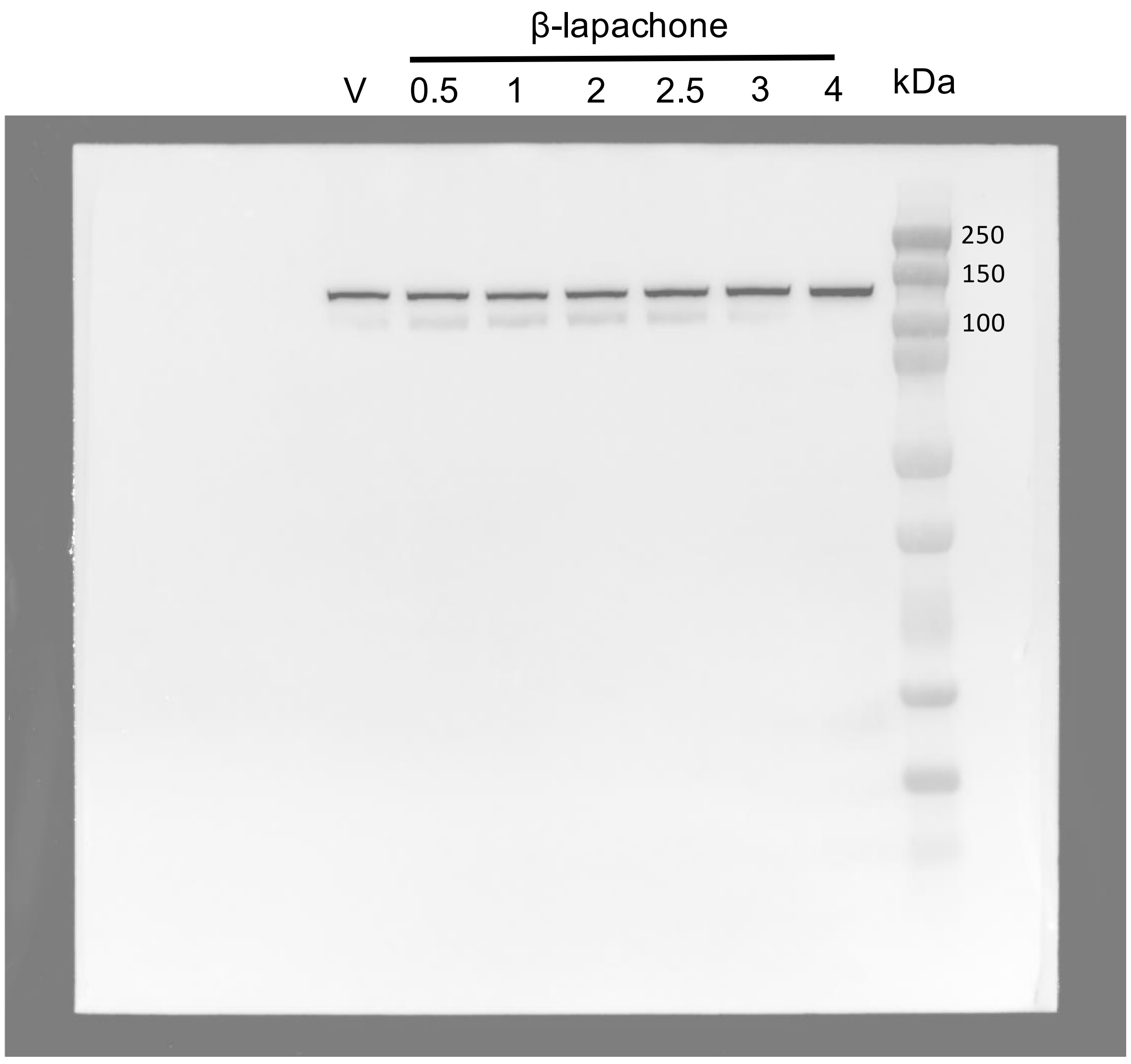
β-lapachone
V 0.5 1 2 2.5 3 4 kDa
250
150
100

Figure 5

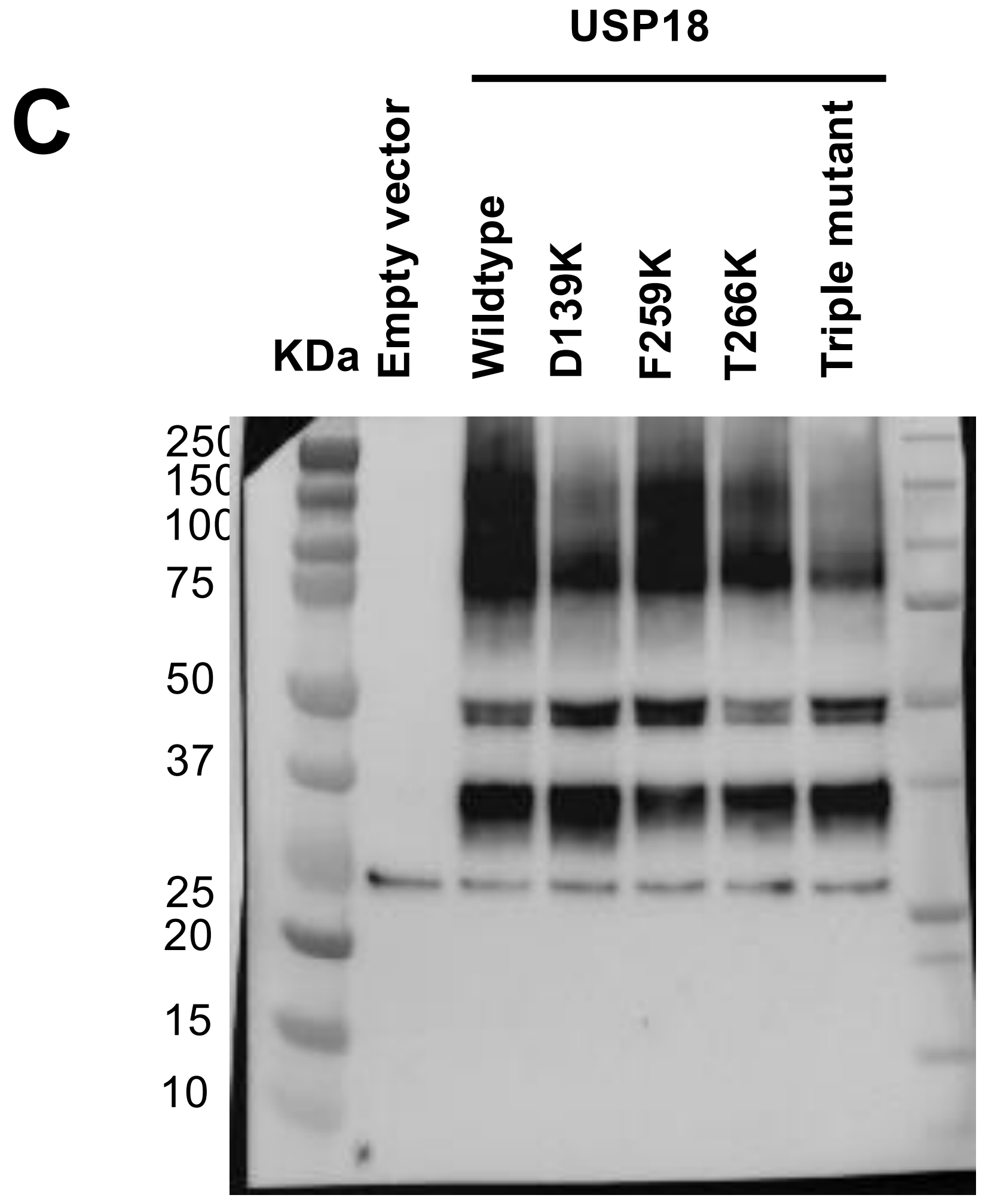
C
USP18
KDa
Empty vector
Wildtype
D139K
F259K
T266K
Triple mutant
250
150
100
75
50
37
25
20
15
10

Figure 7

C

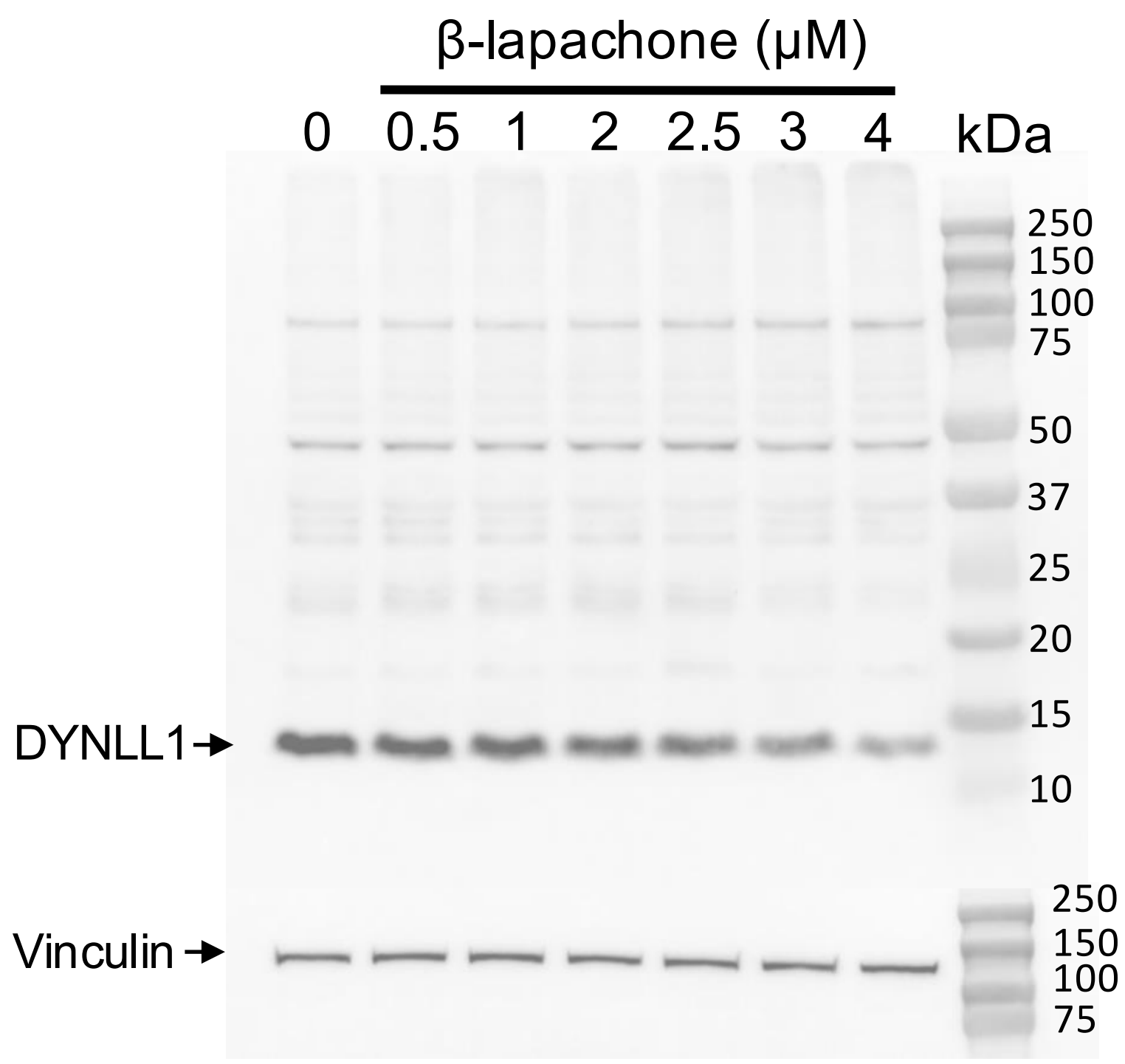

β-lapachone (μM)
0
0.5
1
2
2.5
3
4
kDa
250
150
100
75
50
37
25
20
15
10
DYNLL1
Vinculin
250
150
100
75
